# Effect of acupuncture on the modulation of functional brain regions in migraine: A meta-analysis of fMRI studies

**DOI:** 10.3389/fneur.2023.1036413

**Published:** 2023-03-08

**Authors:** Mengyuan Li, Haipeng Huang, Lin Yao, Hongmei Yang, Shiqi Ma, Haizhu Zheng, Zhen Zhong, Shuo Yu, Bin Yu, Hongfeng Wang

**Affiliations:** ^1^Institute of Acupuncture and Massage, Northeast Asian Institute of Traditional Chinese Medicine, Changchun University of Chinese Medicine, Changchun, China; ^2^Northeast Asian Institute of Traditional Chinese Medicine, Changchun University of Chinese Medicine, Changchun, China; ^3^College of Acupuncture and Massage, Changchun University of Chinese Medicine, Changchun, China; ^4^College of Traditional Chinese Medicine, Changchun University of Chinese Medicine, Changchun, China

**Keywords:** migraine, functional magnetic resonance imaging, acupuncture, meta-analysis, brain regions modulation

## Abstract

**Background:**

Acupuncture, a traditional Chinese medicine therapy, is an effective migraine treatment, especially in improving pain. In recent years, many acupuncture brain imaging studies have found significant changes in brain function following acupuncture treatment of migraine, providing a new perspective to elucidate the mechanism of action of acupuncture.

**Objective:**

To analyse and summarize the effects of acupuncture on the modulation of specific patterns of brain region activity changes in migraine patients, thus providing a mechanism for treating migraine by acupuncture.

**Methods:**

Chinese and English articles published up to May 2022 were searched in three English databases (PubMed, Embase and Cochrane) and four Chinese databases (China national knowledge infrastructure, CNKI; Chinese Biomedical Literature database, CBM; the Chongqing VIP database, VIP; and the Wanfang database, WF). A neuroimaging meta-analysis on ALFF, ReHo was performed on the included studies using Seed-based d Mapping with Permutation of Subject Images (SDM-PSI) software. Subgroup analyses were used to compare differences in brain regions between acupuncture and other groups. Meta-regression was used to explore the effect of demographic information and migraine alterations on brain imaging outcomes. Linear models were drawn using MATLAB 2018a, and visual graphs for quality evaluation were produced using R and RStudio software.

**Results:**

A total of 7 studies comprising 236 patients in the treatment group and 173 in the control group were included in the meta-analysis. The results suggest that acupuncture treatment helps to improve pain symptoms in patients with migraine. The left angular gyrus is hyperactivation, and the left superior frontal gyrus and the right superior frontal gyrus are hypoactivated. The migraine group showed hyperactivation in the corpus callosum compared to healthy controls.

**Conclusion:**

Acupuncture can significantly regulate changes in brain regions in migraine patients. However, due to the experimental design of neuroimaging standards are not uniform, the results also have some bias. Therefore, to better understand the potential mechanism of acupuncture on migraine, a large sample, multicenter controlled trial is needed for further study. In addition, the application of machine learning methods in neuroimaging studies could help predict the efficacy of acupuncture and screen migraine patients suitable for acupuncture treatment.

## 1. Introduction

Migraine is a chronic, primary, intermittent headache disorder characterized by moderate or severe headache attacks, reversible neurological symptoms, and autonomic changes (1). The most typical symptoms include photophobia, vocal intolerance, hyperalgesia on the skin, gastrointestinal discomforts such as nausea and vomiting (2). In addition, migraine patients may have other neurological symptoms such as dizziness, tinnitus, neck pain, depression, and cognitive impairment. Migraine has been ranked by the WHO as one of the most prevalent and second-most disabling neurological disorders worldwide (3), especially in adults between the ages of 25–50. The annual incidence and lifetime prevalence are higher in women than men, at 18 and 33%, respectively (4). Migraines not only affect the quality of life and work efficiency of patients but also bring a tremendous economic burden to society (5).

With the development of molecular biology, genetics and other technologies, people have gradually deepened their understanding of the pathogenesis of migraine. The main mechanisms related to migraine are vascular theory, trigeminal vascular system, neurogenic inflammation, cortical diffusion inhibition and gene inheritance (6, 7). In recent years, the application of brain imaging technology to reveal the pathogenesis of migraine has gradually become an increasing focus of research. Migraine patients present with progressive brain structure and function damage. A voxel-based brain imaging study found that the cingulate cortex, amygdala, insula, frontal lobe and temporal lobe are closely associated with migraine attacks (8). The development of neuroimaging technology provides a new idea for exploring the central pathogenesis of migraine (9). The overuse of drugs is one of the main reasons for migraine, a hot research topic. Medications now commonly used to treat migraine include non-steroidal anti-inflammatory drugs, acetaminophen, ergotamine, migraines' high global disability rate. Therefore, seeking an effective and safe alternative therapy is in line with the requirements of the Healthy China 2030 Plan (10).

Acupuncture is an integral part of traditional medicine and is one of the most common complementary alternatives worldwide, and is widely used in clinical practice in the treatment of migraines. Acupuncture, a traditional Chinese medicine procedure, has been widely used to alleviate diverse types of pain for over 2000 years. In recent years, a growing number of clinical studies have confirmed that acupuncture is the most effective and safe treatment for migraines (11, 12). A meta-analysis of acupuncture for migraines showed that acupuncture had more significant advantages in improving the head frequency of migraine, Visual Analog Scale (VAS), and response rates compared with sham acupuncture (13). Although the application of acupuncture has shown promising efficacy in the clinic, the mechanism of acupuncture still needs to be explored (14).

Neuroplasticity, also known as brain plasticity, is the ability of neural networks in the brain to reorganize and alter structure and function. One of the techniques used to measure neuroplasticity is resting-state functional MRI (rs-fMRI). Because of its simple and easy operation, good spatial resolution, it can locate neurons with spontaneous activity relatively accurately and objectively and is widely used to evaluate the brain network of subjects in a resting state (15). The main analytical methods in rs-fMRI technology include the amplitude of low-frequency fluctuation (ALFF) and regional homogeneity (ReHo) (12). ALFF mainly reflects the brain's inherently low frequencies, while ReHo is used to assess the temporal homogeneity of regional blood oxygen levels dependent on signals. In order to better reveal the intrinsic neural mechanism of action of acupuncture in the treatment of diseases, more and more studies have applied rs-fMRI technology to clinical trials of acupuncture for the treatment of diseases. Since 2008, nearly 30 studies have provided visual evidence of acupuncture treatment for migraine (16). However, due to the different design purposes of the studies, the selected observation area and the sample size are separate, so the imaging evidence for acupuncture treatment of migraine has not yet reached a consensus, which also affects the clinical application of acupuncture to a certain extent.

In recent years, functional neuroimaging studies on the treatment of migraine with acupuncture have increased significantly. These studies are conducive to exploring the plastic regulation of acupuncture on abnormal brain function and functional networks in migraine patients. Some researchers reviewed the neuroimaging research of acupuncture treatment of migraine in recent 10 years from two aspects of the central mechanism of acupuncture prevention and treatment of acute migraine attack and summarized the neuroimaging research status of acupuncture treatment of migraine from two elements of acute analgesia and prevention (17). Another neuroimaging meta-analysis of acupuncture treatment of migraine summarized the published neuroimaging research results of acupuncture treatment of migraine (16). It is concluded that acupuncture can induce different responses in different brain regions of migraine patients, affecting brain activity mainly through regulating pain-related systems. Previous studies mainly summarized the related brain regions descriptively based on the original data. Although it can be concluded that acupuncture has a regulatory effect on the brain regions of migraine patients, it is relatively subjective. Moreover, whether this regulatory effect is related to the intervention of acupuncture on certain specific cerebral regions in migraine patients remains to be further studied.

Therefore, to more objectively explain the neural mechanism of the specific and non-specific effects of acupuncture in the treatment of migraine and clarify which brain regions play an essential role in the treatment of migraine, we applied signed differential mapping (SDM), a new generation of coordinate-based meta-analysis (CBMA) algorithms for analyzing rs-fMRI data from the included study (18). By using the peak coordinates and *t*-values in the included studies, it reassigns the values for each study, is as close as possible to the original research results, tests the heterogeneity, selects the model of random effects, and combines the statistics to obtain coordinate points with statistical differences, which can be analyzed and found in many studies, produce objective, credible and influential research results.

This study aims to use the Seed-based d Mapping with Permutation of Subject Images (SDM-PSI) approach to conduct a meta-analysis of the currently published neuroimaging studies of acupuncture for migraines and to reveal the differential effects of acupuncture treatment on brain regions. The main results summarized in this study may provide new insights and understanding for future studies on brain imaging mechanisms in acupuncture for migraine.

## 2. Materials and methods

We conducted a meta-analysis based on the Cochrane Handbook for Systematic Reviews of Interventions manual. All research procedures follow the recommendations of the Preferred Reporting Items for Systematic Reviews and Meta-Analyses (PRISMA) in Supplementary Table S1. This study is already registered in the Systematic Review Prospective Register (CRD42022323647).

### 2.1. Literature search

Studies included neuroimaging studies about the effect of acupuncture in treating migraine patients. From the inception of the database until May 2022, two independent researchers worked on three English databases (PubMed, Embase and Cochrane) and four Chinese databases (China national knowledge infrastructure, CNKI; Chinese Biomedical Literature database, CBM; the Chongqing VIP database, VIP; and the Wanfang database, WF) for retrieval. The following English search terms were used: “acupuncture,” “fMRI” and “migraine.” The references of all included articles are subjected to secondary examination, and the specific search strategies are supplemented in Supplementary Table S2. The literature search language is limited to Chinese and English, and each database is searched based on its own characteristics.

### 2.2. Inclusion and exclusion criteria

Two researchers searched the entire literature, screened the titles, abstracts, and full texts of the essays, and the following inclusion criteria are formulated according to the PICO standard: (1) Participants: The study was conducted on migraine patients and healthy people of any age and gender, and migraine patients met the diagnostic criteria for migraine (2); (2) Interventions: Only manual acupuncture or electroacupuncture in the treatment group. We have no restrictions on the intensity, frequency or duration of treatment; (3) Comparison groups: The comparison group can be treated with any non-acupuncture method, including sham acupuncture, placebo, basic medication, etc. Meanwhile, health controls are also included as a basis for comparison. But they should be consistent with the baseline information of the intervention group (e.g., age, gender, etc.); (4) Outcomes: The results include functional imaging of the whole brain (ALFF or ReHo) in the resting functional state; The result are present in three-dimensional coordinates (*x, y, z*) reported by the standard stereotactic space Talairach or Montreal Neurological Institute (MNI); If the study involves two or more comparable data, all samples are included (5). Original article, clinical randomized controlled trial; Articles on the study of changes in spontaneous brain activity caused by acupuncture, using rs-fMRI as the primary technical means.

Exclusion criteria: (1) Poor research quality, lack of full text, incomplete and inaccurate literature; (2) Irrelevant articles, reviews, case reports, research protocols, meta-analyses, etc; (3) The study was conducted using only the imaging analysis method of the region of interest (ROI); (4) The sample size for each group of studies was <5.

### 2.3. Data extraction

Extract data from the included literature, form a standard style, mainly containing the year of publication of the document, the name of the first author, the basic information of the participants, For example, migraine type and duration, age and sex, sample size of patients in each group, intervention design (modality of operation and duration of treatment), neuroimaging information (fMRI parameters, coordinates, and *t*-values), secondary outcomes of the study. For the incomplete provision of literature data, get in touch with the corresponding author by email or phone to obtain the data needed for meta-analysis. Data extraction is carried out independently by two members, needs to be cross-checked, and if there is any disagreement, it can be discussed in a targeted manner or resolved according to the opinion of the third part.

### 2.4. Quality assessment

Based on those used in previous neuroimaging meta-analyses, a customized checklist was applied to assess the quality of the included studies in Supplementary Table S3 (19, 20). The checklist included 3 categories and evaluated 12 points, mainly focusing on clinical features, the fMRI data collection and analysis techniques, and the quality of the reported results. We also used the Cochrane Bias Risk Tool, with all reports assessed against the following seven criteria: Random sequence generation, allocation concealment, blinding of subjects and participants, blinding of outcome evaluators, incomplete outcome data, selective reporting of findings and other sources of bias. All the steps were independently performed by two authors, and all inconsistencies were resolved by a third author. The study used *R* software version 4.1.3 and R studio version 2022.02.0 to make visual graphics for quality assessment. Using weighted mean difference (WMD) with 95% CI. The *I*^2^ statistic examined heterogeneity. The forest map was used to show the results of hypothesis testing. If necessary, a sensitivity analysis or subgroup analysis will be conducted.

### 2.5. Data synthesis and meta-analysis

Meta-analysis using Seed-based d Mapping with Permutation of Subject Images (SDM-PSI) (version 6.21, https://www.sdmproject.com/) software, a tutorial on the operation of the division is provided on this website. Firstly, extract the peak coordinates and effect values (*t*-value or *z*-value) of the differential brain regions in various studies (treatment group and control group), and the coordinates were uniformly converted to MNI types by software; Reconstruction of images of discrepant brain regions by software reassigns values to each voxel to maximize similarity to the actual study results. Second, meta-analysis consists of calculating the mean of random effects for ReHo values, with the mean plot weighted by the sample size and variance of each study; A homogeneity test was performed on the included studies using software, which could indicate a pooled effect if the test met *p* > 0.10 and *I*^2^ < 50%; After the SDM-PSI plot reconstruction of each included study, a meta-analysis (mean analysis, displacement test, sensitivity analysis, meta-regression analysis) was performed; All results would be reported using the TFCE-based FWE corrected threshold (*p* < 0.05 and voxel extent ≥10), and obtained voxel clusters with statistically significant differences; Finally, the results were visualized using MRIcronGL software to map the 3D coordinate data to a high-resolution anatomical template. Clinical variables: statistical analyses of continuous data were performed with R software, version 4.1.3 (R Foundation for Statistical Computing, Vienna, Austria), using the Meta and Metafor meta-analysis packages.

### 2.6. Meta-regression analysis

The potential correlation of clinical variables such as VAS scores, mean age, and head frequency were explored by simple linear regression analysis. Linear regression models were plotted using Matlab 2018a.

## 3. Results

### 3.1. Include studies

**Figure 1:**
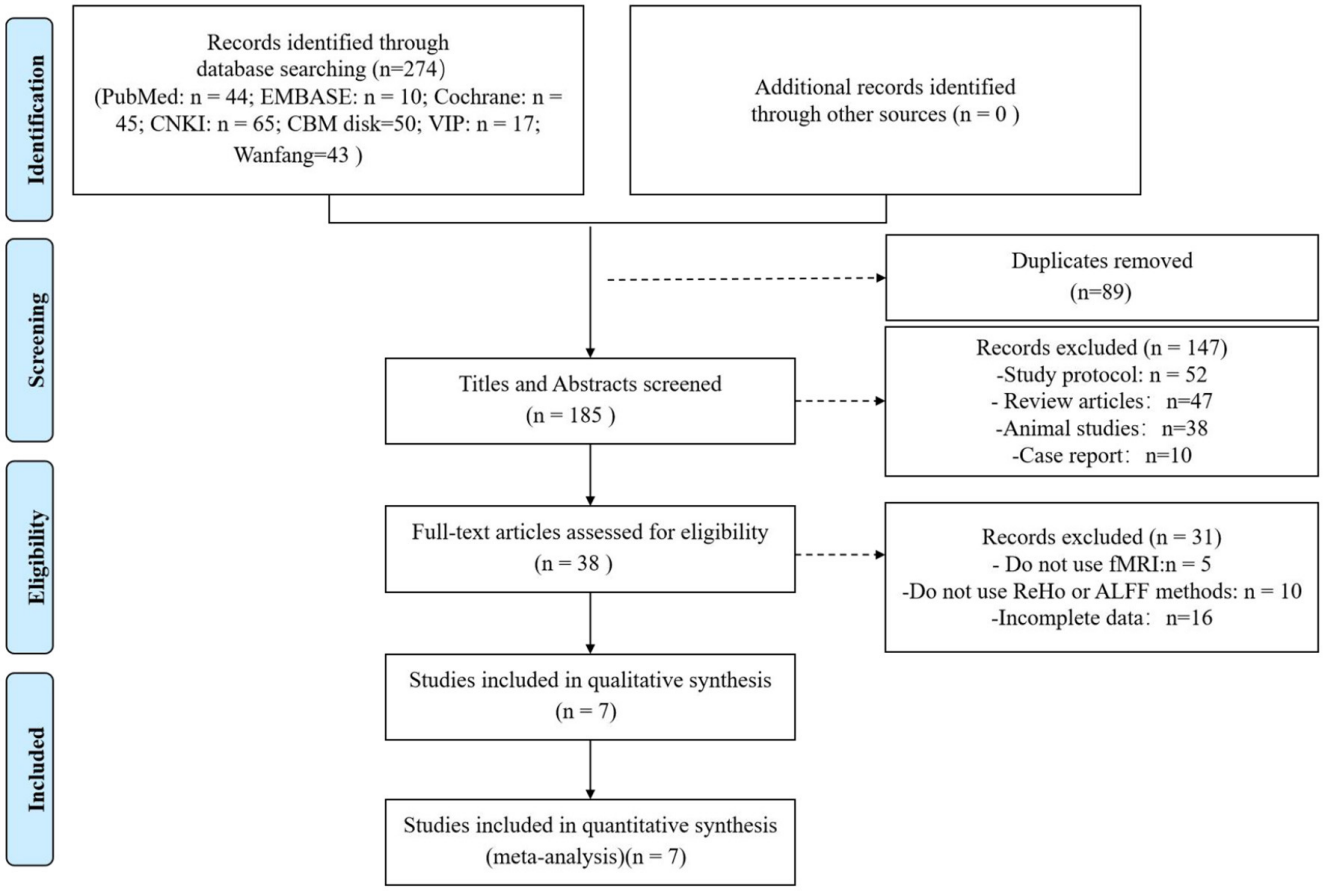
Flowchart of literature selection.

The database search yielded 274 articles according to the search stategy. 89 duplicates were removed, and 185 articles were excluded after screening titles and abstract. Of the 38 potentially relevant reports, 10 articles were did not use ReHo or ALFF, 16 articles did not have complete data, 5 articles did not use fMRI as the primary method. A total of 7 articles proved eligible after full-text screening. 3 of them belong to a longitudinal study (21–23). Acupuncture intervention was performed on migraine patients to reveal the difference in brain regions pre-and-post acupuncture treatment. Figure 1 represents the PRISMA flow diagram of the article search.

Trials were published after 2010, each trial enrolled 10–80 patients, which totally including 443 patients (253 migraine patients and 190 healthy controls), fulfilled the inclusion criteria. All of the 7 included studies were patients with migraine without aura (MwoA). There were no significant differences between the two groups in terms of demographic baseline variables such as age, gender, disease status including symptom duration, or secondary outcome measures. All patients in the treatment group underwent 2–8 courses (weeks) of manual acupuncture therapy. All manual acupuncture method in these trials were empirical acupoints, based on the doctors own clinical experience. All seven studies reported fMRI coordinate data, using ReHo and ALFF variables to analyse the effects of brain region activation before and after the acupuncture intervention and its differences with healthy controls. Clinical efficacy was examined by visual analog scale (VAS) (*n* = 5), self-rating depression scale (SDS) (*n* = 3), self-rating anxiety (SAS) (*n* = 3) and head frequency (*n* = 4). The clinical variables and fMRI data details of the included studies were presented in Tables 1, 2.

**Table 1:** Demographic and clinical characteristics of included studies.

**Study**	**Subtype of migraine**	**Aura**	**Sample size**		**Gender (male/female)**		**Age**		**Intervention**		**Comparison**		
			**Migraine**	**Healthy control**	**Migraine**	**Healthy control**	**Migraine**	**Healthy control**	**Main acupoints**	**Regimen**	**Migraine**	**Healthy control**	**Participant duration**
Han Xiao	Episodic	MwoA	10	10	2/8	2/8	31.7	31.7	MA: GB41(2)	Not reported	Acu	Con	Not reported
Ning Yanzhe	Episodic or chronic	MwoA	19	18	3/16	4/14	28.23	27.16	MA: GB41(2)	Not reported	Acu	Con	A: 77. 68 ± 57. 57
Jia Jingnan	Episodic or chronic	MwoA	15	None	5/10	None	39.3	None	MA: GB44(2), ST45(2), BL67(2), LR1(2)	3/week, for 4 weeks	Acu	None	Not reported
Tao Yin	Episodic or chronic	MwoA	40	40	10/30	10/30	21.7	21.1	MA: SJ(5), GB34(2), GB40(2)	5/week, for 4 weeks	Acu	Con	A: 53.75 ± 28.45
Ling Zhao	Episodic	MwoA	20	20	6/14	8/12	32.90	37.25	MA: SJ5(2), GB20(2), GB34(2), GB40(2)	for 8 weeks	Acu(active)	Acu(inactive)	A: 10.58
Zhengjie Li	Chronic	MwoA	62	42	14/48	8/34	21.21	21.29	MA: ST36(2), ST42(2), LI6(2)	5/week, for 4 weeks	Acu	Con	A: 62.91
Zhengjie Li	Episodic	MwoA	70	43	14/56	9/34	21.51	21.23	MA: GB34(2), GB40(2), SJ5(2)	5/week, for 4 weeks	Acu	Con	A: 61.67

MA, manual acupuncture; Acu, acupuncture; Con, Control; GB41, Zulinqi; GB44, Zuqiaoyin; ST45, Lidui; BL67, Zhiyin; LR1, Dadun; SJ5, Waiguan; GB34, Yanglingquan; GB40, Qiuxu; ST36, Zusanli; ST42, Chongyang; LI6, Pianli.

**Table 2:** Scanning methods in the included studies.

**Study**	**Scanner**	**FWHM**	**Threshold**	**Software**	**Outcome measures**			**Quality score**
					**Analysis of fMRI**	**Coordinate**	**Secondary outcomes**	
Han Xiao	3.0T	Unknown	*p* < 0.05, monte carlo	SPM8	ReHo	Talairach	Not reported	10.5
Ning Yanzhe	3.0T	4	*p* < 0.05, monte carlo	Rest, DPARSF	ALFF	MNI	Not reported	11.5
Jia Jingnan	3.0T	Unknown	*p* < 0.05, GRF	DPARSF	ReHo	MNI	VAS, MSQ, SAS, SDS	11
Tao Yin	3.0T	6	*p* < 0.05	SPM12	ALFF	MNI	VAS, MMDs	11
Ling Zhao	3.0T	4	*p* < 0.05, FDR	SPM5	ReHo	Talairach	VAS, HIT-6	12
Zhengjie Li^1^	3.0T	6	*p* < 0.001, uncorrected; *p* < 0.05, FWE	SPM12	ALFF	MNI	VAS, SAS, SDS	12
Zhengjie Li^2^	3.0T	8	*p* < 0.001, uncorrected; *p* < 0.05, FWE	SPM12	ALFF	MNI	VAS, SAS, SDS	12

ReHo, regional homogeneity; ALFF, amplitude of low-frequency fluctuations; MNI, montreal neurological institute; FDR, false discovery rate; FEW, family-wise error; FWHM, full width at half maximum; VAS, visual analog scale; MSQ, Minnesota satisfaction questionnaire; SAS, self-rating anxiety scale; SDS, self-rating depression scale; MMDs, monthly migraine days; HIT-6, headache impact test version.

### 3.2. Quality assessment

**Figure 2:**
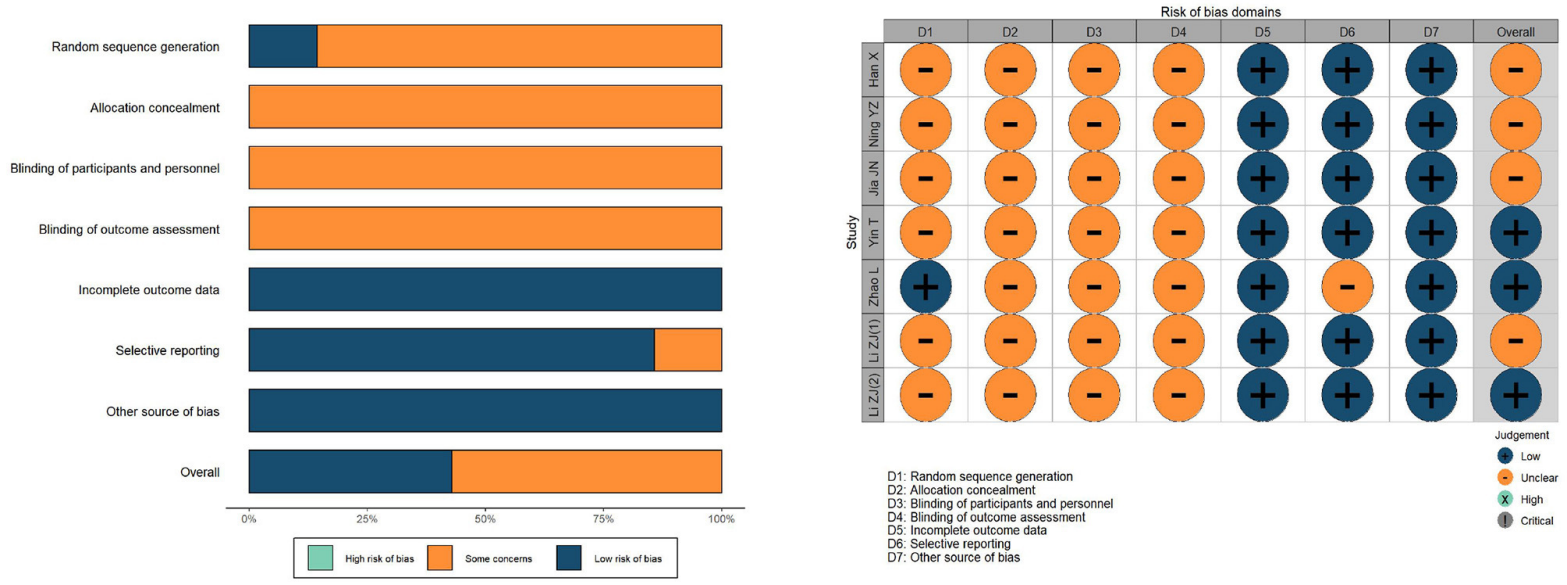
Quality assessment of included studies.

Quality assessment is based on Quality Assessment Checklist and the Cochrane Bias Risk Assessment Tool. In the Quality Assessment Checklist, we assessed the demographic and clinical characteristics of the studies, sample size, MRI scanner parameters, analytical techniques and the quality of the reported results, with all seven studies scoring >10. In the Cochrane Bias Risk Assessment Tool, only 1 reported the method of generating random sequences (21). All studies have not mentioned the allocation concealment and blind method, and the bias risk is unclear. The evaluation of data for incomplete results depended on whether the baseline data in the studies were clearly shown and all studies included were at low risk. Although none of the studies had a clear study protocol, all expected outcome indicators were reported, including predetermined ones. We prefer to consider studies with two or more secondary outcome indicators reported to be at low risk for selectively reported data (24). Therefore, six studies had a low risk of bias in selective reporting, except Zhao's research (21). In addition, we found no other sources of bias. Overall, the quality of the included studies was poor, mainly in allocation concealment, blinding and blinding of outcome assessment. Figure 2 shows the quality assessment of the 7 included studies.

### 3.3. Meta-analysis of brain region changes

#### 3.3.1. The effect of acupuncture on the modulation of brain regions in migraine patients

**Figure 3:**
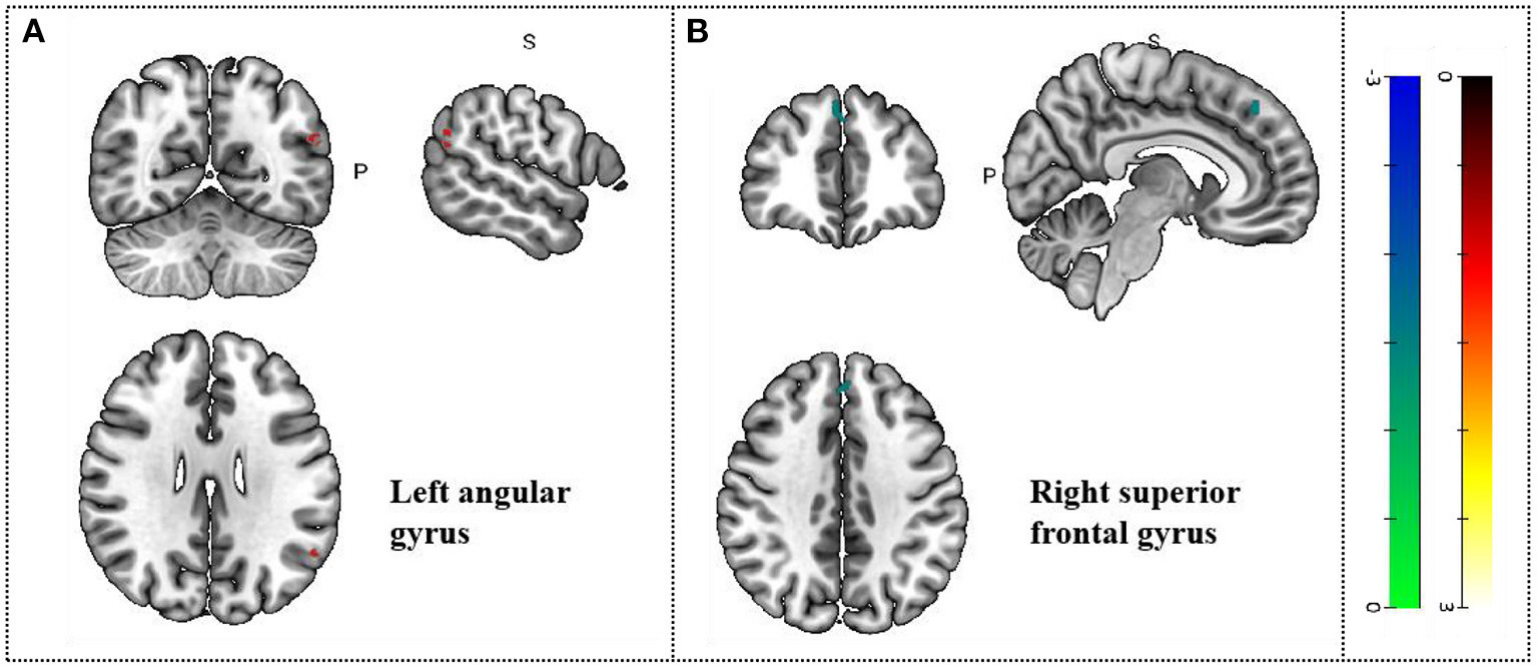
Changes in gray matter regions PRE and POST acupuncture treatment in MCI patients. **(A)** Left angular gyrus. **(B)** Right superior frontal gyrus. Important clusters are presented with MRIcron templates.

Comparison with pre-acupuncture treatment, patients in post-acupuncture showed hyperactivation in the left angular gyrus (*p* < 0.05, *z* = 5.518); And the decreased activity of the right superior frontal gyrus, medial, BA 8 (*p* < 0.05, *z* = −3.853). Peak coordinates and cluster breakdowns are shown in Table 3. Differences in the brain regions between pre-and-post acupuncture treatment have been visualized in Figure 3.

**Table 3:** Brain activity changes in patients PRE and POST acupuncture treatment in the coordinate-based meta-analysis.

**Brain regions**	**MNI coordinates**			**SDM *z*-score**	***p*-value**	**Voxels**	**Cluster breakdown (number of voxels)**	***I* ^2^**
	***x***	***y***	***z***					
L angular gyrus, BA 22	−58	−60	24	5.518	0.047999978	21	Left angular gyrus, BA 39 (14)	2.544912
							Left angular gyrus, BA 22 (11)	
R superior frontal gyrus, medial, BA 8	6	38	44	−3.853	0.041999996	35	Left superior frontal gyrus, medial, BA 9 (24)	10.513831
							Right superior frontal gyrus, medial, BA 9 (19)	
							Right superior frontal gyrus, medial, BA 8 (15)	

Peak height threshold: z > 1; Voxel probability threshold: after correcting threshold (TFCE) of p < 0.05; Cluster extent threshold: number ≥10 voxels; BA, Brodmann area; I^2^, heterogeneity I^2^; MNI, montreal neurological institute; L, left; R, right; ReHo, regional homogeneity; SDM, signed differential mapping.

#### 3.3.2. Differences in brain region between migraine patients and healthy controls

**Figure 4:**
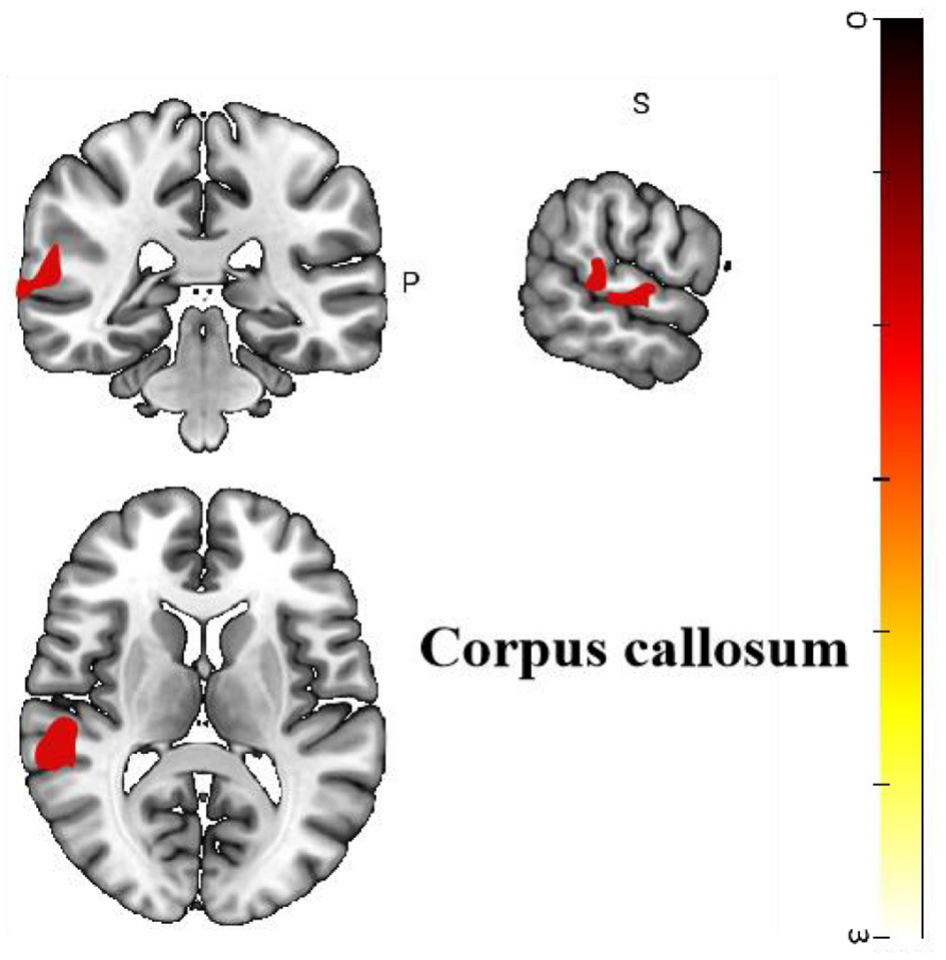
Regional differences in gray matter between migraine patients and healthy controls before acupuncture treatment.

Four cross-sectional studies compared the different brain regions between healthy controls and migraine patients (25–28). The results showed that compared with healthy controls, migraine patients in the treatment group showed hyperactivation in the corpus callosum (*p* < 0.05, *z* = 3.665). The right superior temporal gyrus also showed activation. Differences in the brain regions between healthy controls and migraine patients have been shown in Table 4 and Figure 4.

**Table 4:** Brain activity changes in migraine patients vs. healthy controls in a coordinate-based meta-analysis.

**Brain regions**	**MNI coordinates**			**SDM *z*-score**	***p*-value**	**Voxels**	**Cluster breakdown (number of voxels)**	***I* ^2^**
	***x***	***y***	***z***					
Corpus callosum	62	−18	4	3.665	0.029999971	103	Corpus callosum (35)	13.584920
							Right superior temporal gyrus, BA 22, (32)	
							Right superior temporal gyrus, BA 21, (24)	
							Right superior temporal gyrus, BA 48, (15)	
							Right superior temporal gyrus, BA 42, (14)	
							Right superior longitudinal fasciculus III, (10)	

Peak height threshold: z > 1; Voxel probability threshold: after correcting threshold (TFCE) of p < 0.05; Cluster extent threshold: number ≥10 voxels; BA, Brodmann area; I^2^, heterogeneity I^2^; MNI, Montreal Neurological Institute; ReHo, regional homogeneity; SDM, signed differential mapping.

### 3.4. Heterogeneity analysis and publication bias

Analysis of heterogeneity revealed variability and diversity among the included studies. Among all 7 studies, the angular gyrus, the left superior frontal gyrus, the right superior frontal gyrus and the corpus callosum showed low between-study heterogeneity of effect size differences in peak coordinate (*I*^2^ = 2.54–13.58%). Tables 3, 4 shows the heterogeneity results. In these studies, the Egger tests had insignificant (*p* = 0.649), and there were no significant publication bias.

### 3.5. Meta regression analysis

**Figure 5:**
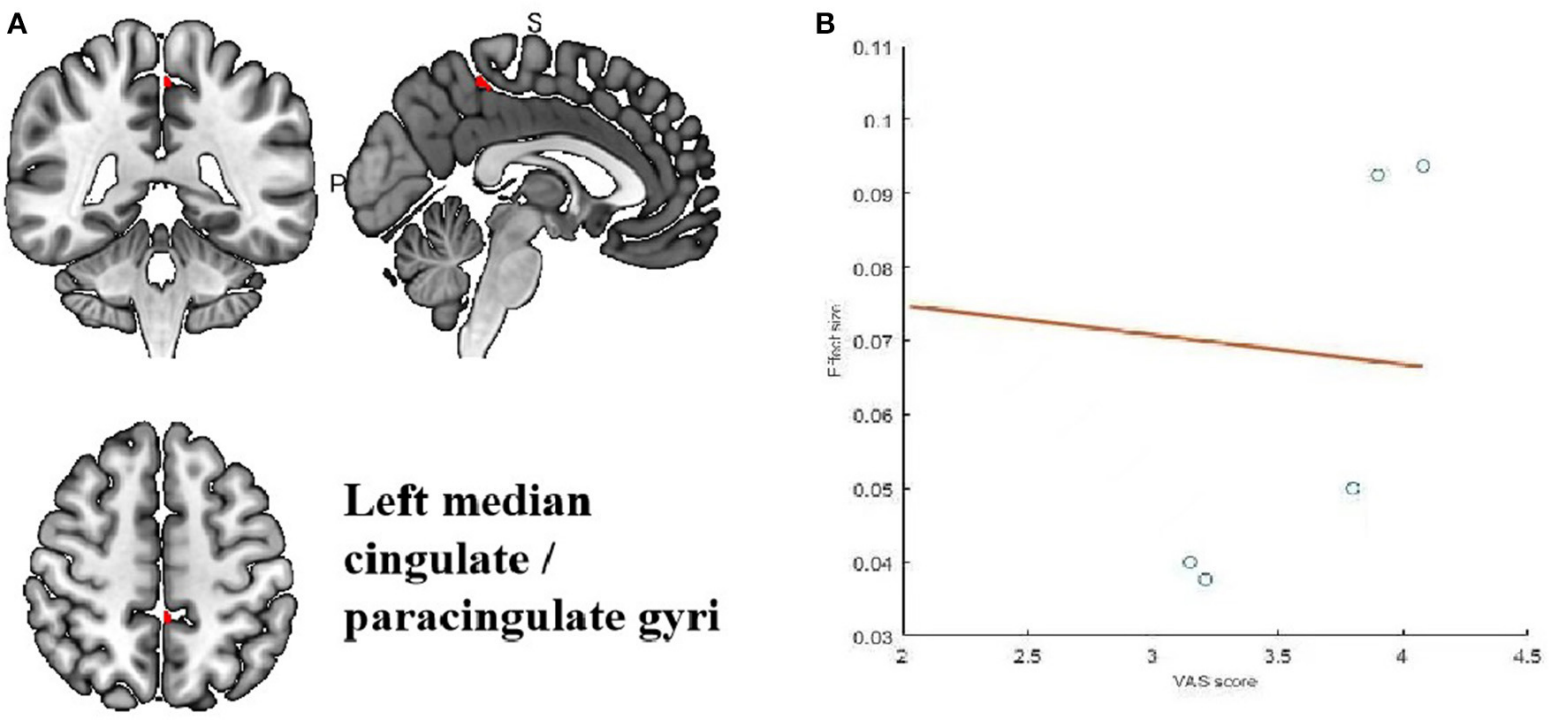
Results of Meta-regression linear model analysis. **(A)** VAS scores of migraine patients are positive correlated with regional activity in the Left median cingulate/paraxingulate gyri. **(B)** The effect sizes needed to create this plot were extracted from the peak voxels of the maximum slope difference. All studies are indicated by the empty blue circles. Regression lines (Meta-regression SDM slopes) are shown as straight lines.

**Figure 6:**
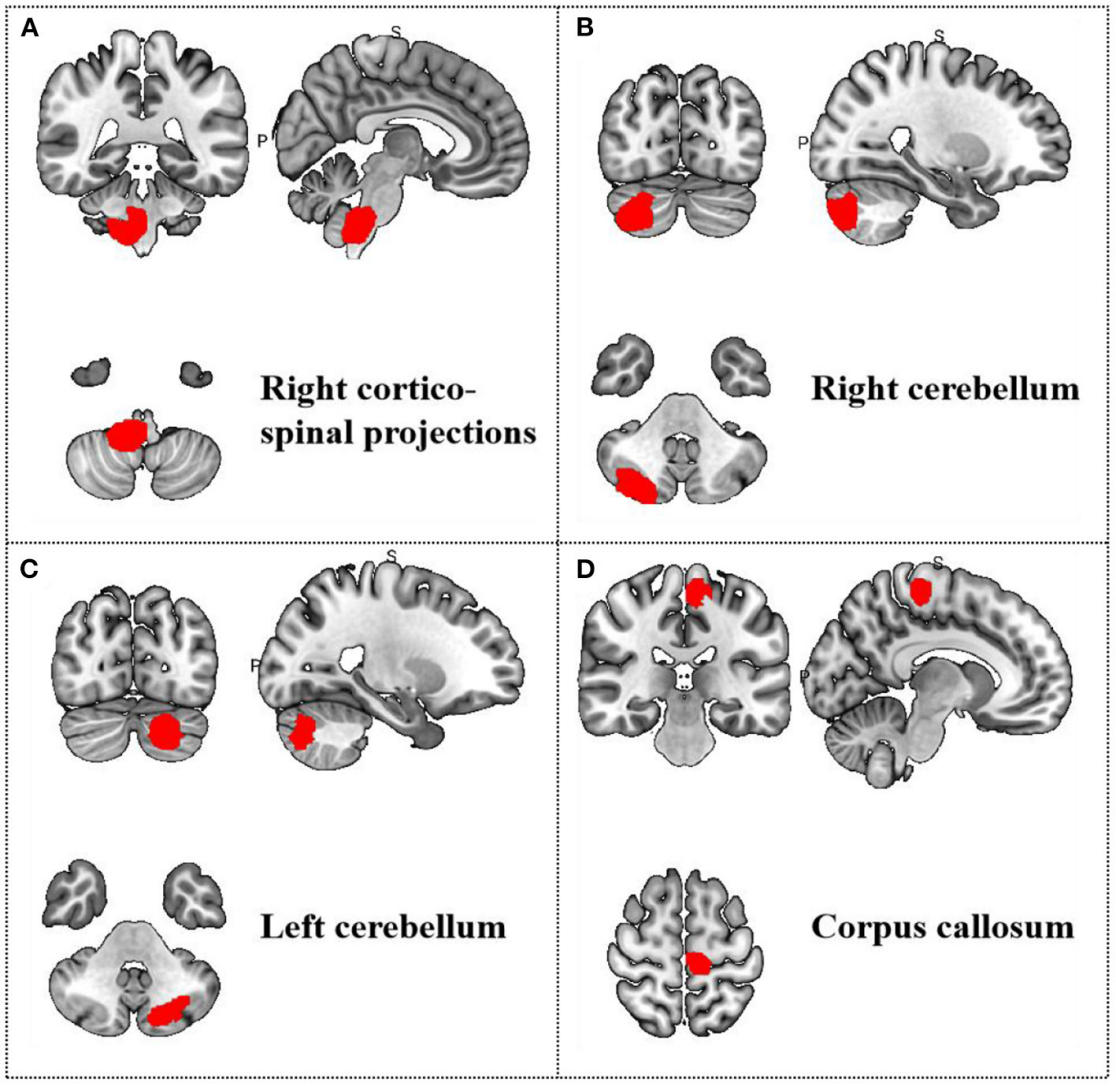
Results of Meta-regression linear model analysis. Age of migraine patients are positive correlated with regional activity in **(A)** the right cortico-spinal projections, **(B)** the right cerebellum, **(C)** the left cerebellum, and **(D)** the corpus callosum.

**Figure 7:**
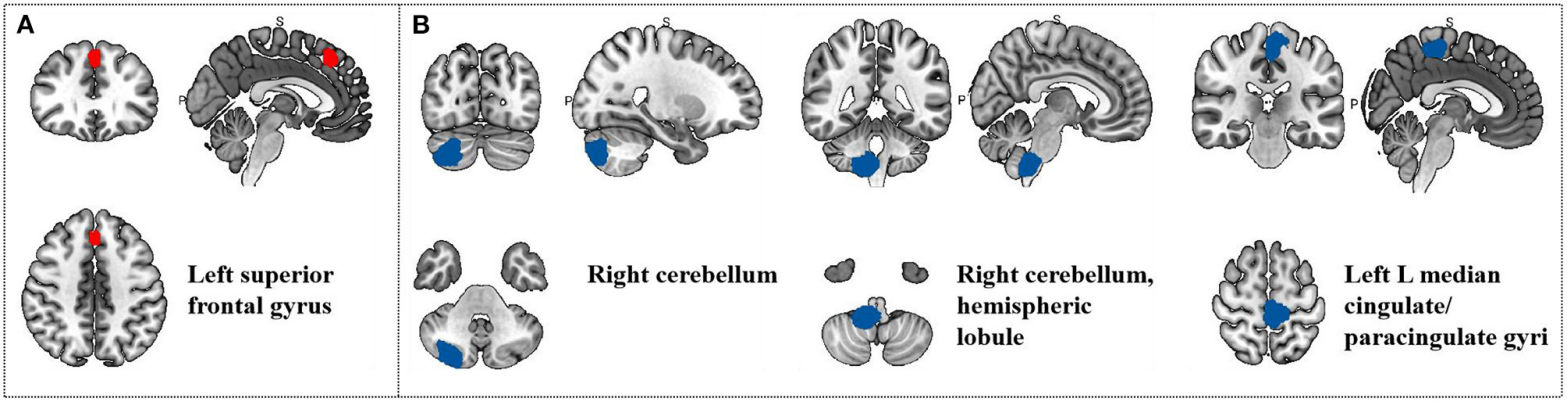
Results of Meta-regression linear model analysis. **(A)** Head frequency of migraine patients are positive correlated with regional activity in the Left superior frontal gyrus. **(B)** Head frequency of migraine patients are negative correlated with regional activity in the Right cerebellum, the Right cerebellum, hemispheric lobule and the Left median cingulate/paracingulate gyri.

**Figure 8:**
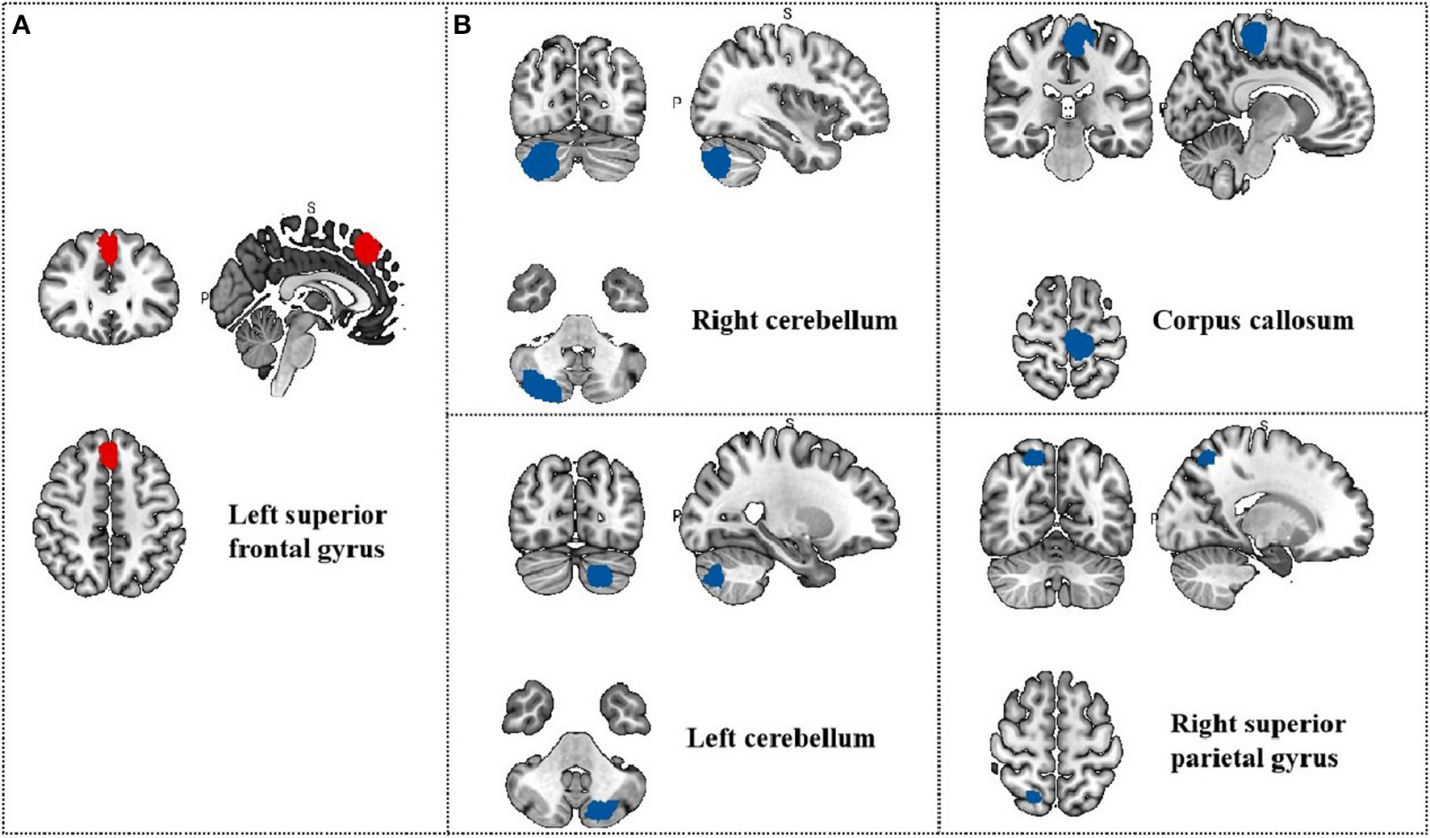
Results of Meta-regression linear model analysis. **(A)** SAS scores of migraine patients are positive correlated with regional activity in the Left superior frontal gyrus; **(B)** SAS scores of migraine patients are negative correlated with regional activity in the Right cerebellum, the Left cerebellum, the Corpus callosum and the Right superior parietal gyrus.

Meta-regression was used to search for the potential correlation between baseline information, subjective scales and brain regions. The meta-regression analysis showed that VAS scores in migraine patients were positive correlated with brain region activity in the left median cingulate/paracingulate gyri in Table 5 and Figure 5. The mean age are associated with the right cortico-spinal projections, right cerebellum, crus II, left cerebellum, crus II and corpus callosum in Table 6 and Figure 6. The head frequency is associated with many regions, including the left superior frontal gyrus, the right cerebellum, crus II, the right cerebellum and the median cingulate/paracingulate gyri in Table 7 and Figure 7. Only one subjective scale showed obvious correlation with brain regions changes. The SAS scores in migraine patients were positive correlated with brain region activity in the left superior frontal gyrus and negative correlated with regional activity in the right cerebellum, the left cerebellum, the corpus callosum and the right superior parietal gyrus in Table 8 and Figure 8. There were no significant correlation between any changes of brain functional regions and gender percentage.

**Table 5:** Meta-regression analysis of VAS scores in treatment group.

**Region**	**MNI coordinate**			**SDM *z*-score**	***p*-value**	**Number of voxels**
	***x***	***y***	***z***			
L median cingulate/paracingulate gyri	−2	−40	54	3.150	0.004999995	30

Peak height threshold: z > 1; Voxel probability threshold: p < 0.05; Cluster extent threshold: number ≥10 voxels; MNI, Montreal Neurological Institute; L, left; SDM, signed differential mapping.

**Table 6:** Meta-regression analysis of mean age in treatment group.

**Region**	**MNI coordinate**			**SDM *z*-score**	***p*-value**	**Number of voxels**
	***x***	***y***	***z***			
R cortico-spinal projections	8	−38	−38	3.436	0.004999995	1156
R cerebellum, crus II	30	−86	−44	2.894	0.004999995	1067
L cerebellum, crus II	−22	−82	−40	2.874	0.004999995	752
Corpus callosum	−10	−28	60	2.669	0.004999995	311

Peak height threshold: z > 1; Voxel probability threshold: p < 0.05; Cluster extent threshold: number ≥10 voxels; MNI, Montreal Neurological Institute; L, left; R, right; SDM, signed differential mapping.

**Table 7:** Meta-regression analysis of head frequency in treatment group.

**Region**	**MNI coordinate**			**SDM *z*-score**	***p*-value**	**Number of voxels**
	***x***	***y***	***z***			
L superior frontal gyrus, medial, BA 8	0	30	50	4.217	0.004999995	237
R cerebellum, crus II	30	−84	−42	−3.337	0.004999995	1091
R cerebellum, hemispheric lobule IX	12	−42	−52	−3.623	0.004999995	985
L median cingulate / paracingulate gyri	−4	36	52	−3.659	0.004999995	1016

Peak height threshold: z > 1; Voxel probability threshold: p < 0.05; Cluster extent threshold: number ≥10 voxels; MNI, Montreal Neurological Institute; L, left; R, right; SDM, signed differential mapping.

**Table 8:** Meta-regression analysis of SAS in treatment group.

**Region**	**MNI coordinate**			**SDM *z*-score**	***p*-value**	**Number of voxels**
	***x***	***y***	***z***			
L superior frontal gyrus, medial	0	30	46	3.724	0.004999995	686
R cerebellum, crus II	30	−88	−38	−3.823	0.004999995	1700
Corpus callosum	−8	−28	60	−3.728	0.004999995	1107
L cerebellum, crus II	−22	−82	−40	−3.661	0.004999995	583
R superior parietal gyrus, BA7	20	−62	58	−3.000	0.004999995	253

Peak height threshold: z > 1; Voxel probability threshold: p < 0.05; Cluster extent threshold: number ≥10 voxels; MNI, Montreal Neurological Institute; L, left; R, right; SDM, signed differential mapping.

### 3.6. Meta-analysis of secondary outcomes

**Figure 9:**
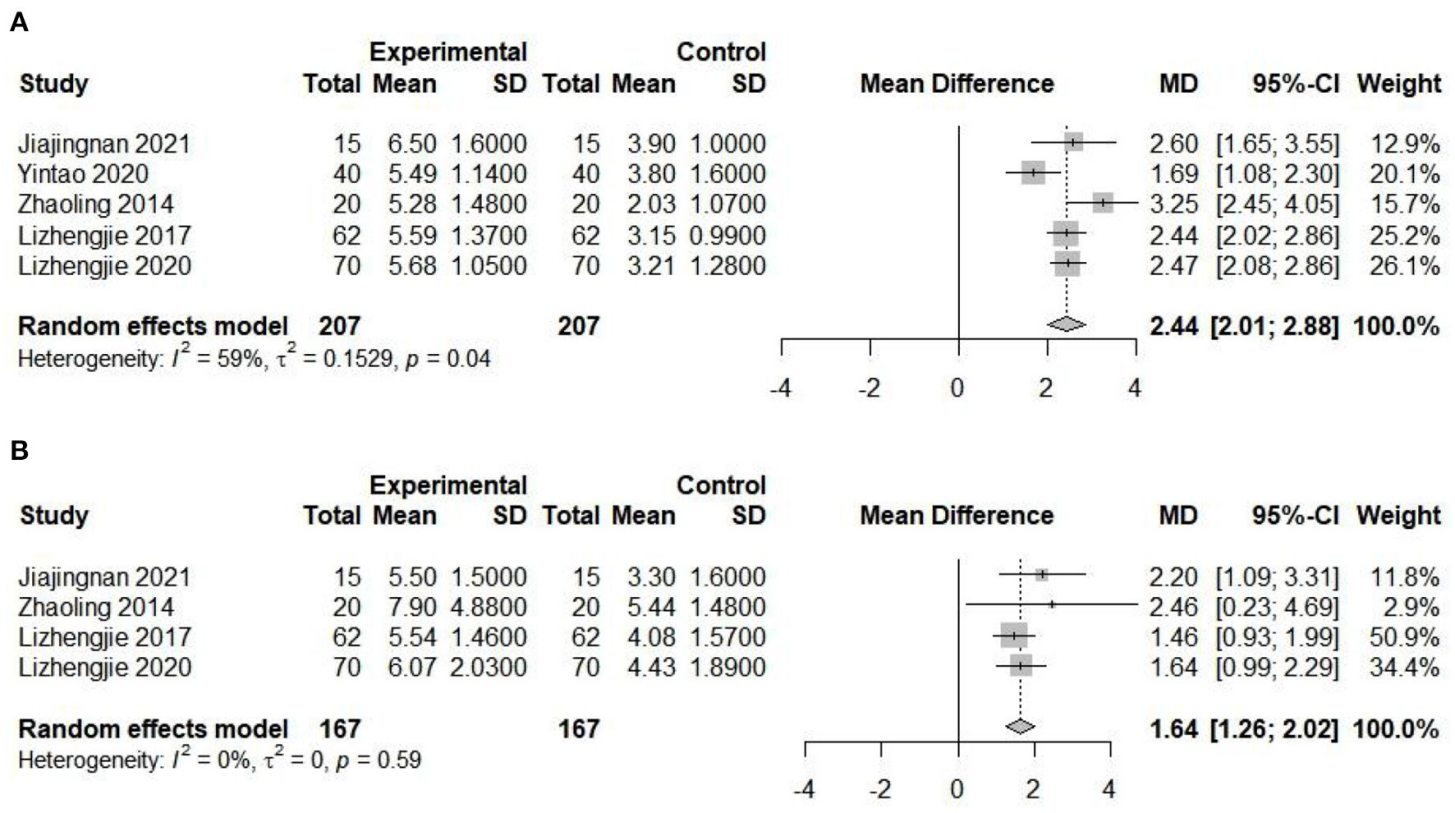
Forest plot presenting the study meta-analysis for the **(A)** VAS scale. **(B)** head frequency.

Manual acupuncture therapy was led to a lower VAS scores when compared to pre-acupuncture (2.44; 95% CI, 2.01–2.88; *I*^2^ = 59%), indicating that acupuncture could relieve the patients' painful symptoms. The change in head frequency was also decrease (1.64; 95% CI, 1.26–2.02; *I*^2^ = 0%), it has been shown that acupuncture can relieve the frequency of headache attacks in migraine patients. The forest plots are shown in Figures 9A, B.

## 4. Discussion

With the widespread use of brain imaging techniques in the field of acupuncture effect mechanisms, there has been an increasing number of studies using fMRI techniques to reveal the central effect mechanisms of acupuncture in the treatment of migraine. In this study, we used the SDM-PSI method to conduct a meta-analysis of the brain imaging data of migraine patients treated with acupuncture and summarized the regulatory effect of acupuncture on migraine brain regions from the neuroimaging perspective. The results of the within-group comparison confirmed the effect of acupuncture on the regulation of brain regions in migraine patients, with activation in the left angular gyrus, left superior frontal gyrus and right superior frontal gyrus after acupuncture treatment. Meanwhile, the comparison between groups showed significant differences in the areas of brain activation in the migraine group after acupuncture treatment compared to healthy controls, mainly in the corpus callosum. In addition, the left cingulate gyrus correlated significantly with VAS scores; the right cortico-spinal projections, left cerebellum, and right cerebellum correlated with the mean age of migraine patients; and the left superior frontal gyrus, right cerebellum, and left cingulate gyrus correlated significantly with headache frequency. Functional features show that these regions are mainly involved in motor, pain, cognitive, control and decision-making. From a neuroimaging perspective, this may provide a possible mechanism for acupuncture in the treatment of migraine.

### 4.1. Study and experimental design

Of the original studies, we included, only 1 was an RCT. Randomized controlled trials (RCTs) are considered the gold standard for evaluating medical research and allow causal inferences to be made about the effects of the interventions studied (29). Due to sample size limitations in MRI trials, the number of subjects is often limited by practical constraints such as scan time, resulting in a small sample size of subjects included in the study, which in turn prevents a large scale RCT. therefore, larger sample size RCTs should be considered in the design of future clinical trials related to acupuncture. In the original studies, two were task-based fMRI (Tb-fMRI) and five were resting-state fMRI. Rs-fMRI or task-free fMRI requires subjects to be in the “default” or “idle” state as much as possible, whereas Tb-fMRI collects signal changes triggered by pre-designed tasks or passive stimuli (30). Current research on the mechanisms of potential central effects of acupuncture also falls into these two main categories. The rs-fMRI studies of acupuncture usually aim to explore the long-time effects of acupuncture by observing the changes in BOLD before and after a course of acupuncture treatment. Tb-fMRI studies induce precise activation of the brain through a predetermined acupuncture activity or task with the aim of exploring the immediate effects of acupuncture. the most common design of Tb-fMRI is the block design task. Because of the transient nature of pain, Tb-fMRI studies are more relevant than rs-fMRI for exploring the analgesic mechanisms of acupuncture (31). For the data analysis mode of MRI, the included original studies mainly used Reho or Alff for data analysis. Reho mainly describes the similarity of the time series of a given voxel (BOLD signal) with that of its nearest neighbor (BOLD). ALFF, on the other hand, reveals the BOLD signal intensity of regional spontaneous activity. Observing the effects of acupuncture on functional brain activity by combining the results of both analysis methods enables more accurate and comprehensive conclusions to be drawn.

In all included studies, in addition to applying brain imaging techniques to observe the modulation of specific brain regions by acupuncture in migraine patients, subjective scales were likewise used for evaluation. VAS scales is used to measure the intensity of pain perceived by the patient (32). The scale has been proved to be a reliable and effective measurement of pain intensity and is sensitive to clinical changes in pain (33). Of all 7 included studies, except for 2 which did not report secondary outcomes, the VAS scale was used to measure patients' subjective pain. Although the scale is a vital evaluation method in clinical trials, the disadvantage is that patients are too subjective to make the results biased. Therefore, in future studies, we should explore an objective test for the evaluation of migraine, combining the subjective and objective, so that we can better evaluate migraine patients.

### 4.2. Characteristics of acpoints selection in acupuncture for migraine

Manual acupuncture (MA) was chosen as the primary treatment for migraine in all 7 of the included studies. MA is a standard method of acupuncture used by acupuncturists and is very effective in relieving pain (34). In addition to inserting acupuncture needles into specific acupoints, manual operation of the needle (intermittent rotation and lifting and inserting) can improve its clinical benefit. As can be seen from the main acupuncture points selected for the included studies, most of them are located on the Gallbladder Meridian of Foot-Shaoyang and the Sanjiao Meridian of Hand-Shaoyang. Migraine mainly involves the orbit, forehead and other parts, which is consistent with the route of the Sanjiao Meridian and Gallbladder Meridian in the head. GB40 is located in the front and bottom of the ankle of the foot, and GB34 is located on the lateral side of the lower leg; they both belong to the point on the Gallbladder Meridian of Foot-Shaoyang. SJ5 belongs to the Sanjiao Meridian of Hand-Shaoyang and is located on the wrist two inches above the transverse wrist line, in the space between the ulna and the radius. Recent studies have shown that pain processing is abnormal in the brain's frontal, parietal and limbic regions in migraine patients and that acupuncture of GB34, GB40, and SJ5 can restore pain processing and modulate pain perception (35). Two studies used only one acupuncture point GB41 for treatment, while others combined multiple acupuncture points for migraine. GB41 is an essential acupoint for migraine. Acupuncture GB41 increases the functional connectivity of pain-related brain areas, which are primarily located in the nociceptive transmission pathways and somatosensory cortex, and are the main nociceptive modulation centers (36). Our study found that the duration of acupuncture treatment was inconsistent across studies, which could be related to the experimental design, the acupuncturist's practice and the patient's actual condition. It also suggests that we should follow the Standard Protocol Items: Recommendations for Interventional Trials (SPIRIT) (37) when designing clinical trials in the future to standardize clinical trial practice.

### 4.3. Modulatory effects of acupuncture on brain regions of migraine

Chronic pain is a serious and common global medical problem and is considered to be a central nervous system disorder associated with the activity of multiple networks of the central nervous system (CNS) (38, 39). As a result of CNS multi-network activation, chronic pain consists of multiple components, including sensory, emotional, cognitive and behavioral elements (40). In recent years, a growing number of brain imaging studies have provided important insights into the brain mechanisms underlying migraine. Migraine appears to be a specific model that is involved in the network and underlying mechanisms of brain processing of pain signals, while being associated with sensory, emotional, autonomic and cognitive (41, 42). For example, abnormalities in pain regulation in brain regions such as hypothalamus, brainstem, periaqueductal gray, and raphe magnus (43, 44). In recent years, numerous studies have demonstrated the clinical efficacy of acupuncture for migraine from a neuroimaging perspective (45–47). Acupuncture has been shown to relieve headache symptoms in two ways: by restoring pain processing function and by modulating pain perception (35).

Our meta-analysis found that patients after acupuncture exhibited hyperactivity of the angular gyrus (AG) compared with those before acupuncture treatment. Migraine patients in the treatment group also showed activation in the supra-callosal and right superior temporal gyrus compared to healthy controls. The AG is located at the junction of the occipital, temporal, and parietal lobes and is thought to be an important interface for transmitting and integrating information between different modalities and processing subsystems (48). The angular gyrus is thought to be a common hub connecting pain networks and has been shown to affect chronic pain (49–51). Previous fMRI studies found a significant increase in gray matter volume in the angular gyrus of migraine patients, which may be related to self-adaptation of the central nervous system, leading to abnormal sensitization of the brain (52). In a previous resting-state MRI study of acupuncture in patients with migraine without aura (MwoA), it was found that after 12 acupuncture sessions, the angular gyrus ReHo values increased significantly in the migraine group, which is consistent with the findings of the present study (17). The above study suggests that long-term acupuncture treatment may alleviate the painful symptoms of migraine by modulating the horn back modulation associated with pain and emotions.

The corpus callosum is the main fiber network connecting the cerebral hemispheres and plays a key role in establishing communication about sensory integration, inhibition and attention. It was found that an increase in the size of the anterior corpus callosum was positively correlated with the ability to control pain (53). A fMRI study on the effects of acupuncture on pain-emotion-related brain regions in patients with cervical spondylosis showed that the functional connectivity of the patient's left corpus callosum to the insula was reduced after acupuncture (47). In contrast, our study found that migraine patients in the treatment group showed hyperactivation of the corpus callosum compared to healthy controls. This may suggest that the modulation of pain by acupuncture may act through the corpus callosum, but more clinical studies are needed to demonstrate this.

Recent preliminary data from the laboratory suggest a role for the encoding of experimental pain memory in the superior temporal gyrus (STG) (54). In a randomized controlled trial of the effects of long-term acupuncture on resting-state brain activity in migraine patients, results showed that migraine patients in the active acupuncture point group exhibited significantly higher temporal superior gyrus (STG) ReHo values, again consistent with the results of the meta-analysis in this study (21). From the above findings, we can conclude that acupuncture points may achieve relief of migraine symptoms and promote the establishment of pain homeostasis by modulating key regions and pain circuits such as angular gyrus, corpus callosum, and right superior temporal gyrus.

### 4.4. Strengthens and limitations

This review is special and innovative. Our innovative application of SDM-PSI to the rs-fMRI randomized controlled clinical trial of acupuncture for migraine provides new insights into how acupuncture modulates altered brain activity and central brain effects in migraine patients. In addition, this study developed strict inclusion and exclusion criteria to minimize selection bias and finally conducted a comprehensive analysis of 7 studies. These clinical trials have similar participants and research designs, and the results are comparable.

This study also has certain limitations. The main limitation was the small sample size of the included studies. Only 7 clinical trials and 443 participants met the inclusion criteria, resulting in no publication bias and subgroup analysis of secondary outcome indicators. The lack of high-quality controlled clinical trials makes it difficult to accurately analyse the interaction between acupuncture efficacy and central neuroplasticity in the brain of migraine patients. Therefore, to better understand the potential impact of acupuncture on the plasticity of the migraine brain, it is necessary to organize multi-center, large samples and design a complete randomized clinical trial of acupuncture brain imaging. In addition, different acupuncture parameters, including the choice of acupuncture points, duration of acupuncture treatment, needle manipulation, frequency of needling, and whether or not De qi is obtained, can lead to subtle changes in the structural and functional activity of the brain. Therefore, there may be some heterogeneity in the acupuncture treatments between the included studies. Due to the specificity and diversity of acupuncture treatment, a comprehensive study of the factors influencing acupuncture manipulation and manipulation practices is of great importance.

### 4.5. Future outlook

FMRI is a non-invasive, high-resolution test that indirectly reflects the state of neural activity in the brain based on changes in blood oxygen levels. The advantage is that *in vivo* detection of brain activity is possible, and the construction of complete functional imaging of brain activity (55). In recent years, the combination of acupuncture interventions and neuroimaging has provided objective and visual evidence to reveal the pathogenesis and treatment of migraine (56). In addition to fMRI, positron emission tomography (PET) is also commonly used to identify brain metabolic levels in migraine patients. A voxel-based Fludeoxyglucose PET shows differences in brain metabolism between migraine patients and healthy people. These differential brain regions may have abnormal brain metabolism at the beginning of a migraine attack and corresponding changes after repeated attacks (57). Recently, fMRI-based functional brain connectomics, which includes the entire human brain, has shown that the brain is highly organized with a network of interacting and connected regions and is emerging as a hot spot for research on acupuncture mechanisms (58). These studies have led to a better understanding of the specific changes between the brain and disease, providing new insights into the mechanisms of acupuncture action. Tu et al. conducted a connectome-based fMRI study to identify and validate markers of imaging function for MwoA (59). This marker not only has the potential to diagnose migraine but also to help establish objective and personalized treatment protocols. Recently, Yang et al. developed a model for predicting the efficacy of acupuncture in migraine patients based on pre-processed gray matter structures using a machine learning approach. The model was 83% accurate in distinguishing between those who responded and those who did not respond to acupuncture (60). The above studies suggest that neuroimaging techniques can evaluate and predict clinical efficacy. In future research, we need multimodal neuroimaging studies that combine multiple data analysis methods such as big data, artificial intelligence and machine learning to comprehensively analyse and cross-validate neuroimaging results to reveal the neuroimaging mechanisms of acupuncture for migraine.

## 5. Conclusion

A meta-analysis of the results of 7 rs-fMRI clinical studies of acupuncture for migraine showed that, compared to pre-treatment baseline, the left angular gyrus, left superior frontal gyrus and right superior frontal gyrus may be the precise brain region response targets of acupuncture for migraine. The migraine group showed hyperactivation in the corpus callosum after acupuncture treatment compared to healthy controls. The changes in these brain regions were significantly associated with pain, cognition, vestibular and visual network functions. Our findings will help reveal the central nervous mechanism of acupuncture in the treatment of migraine, provide new insights into the effectiveness of acupuncture, and enable the development of individualized treatment plans for migraine patients.

## Data availability statement

The original contributions presented in the study are included in the article/Supplementary material, further inquiries can be directed to the corresponding author.

## Author contributions

ML and HH designed the entire study and wrote the manuscript. LY, HY, and SM screened the study for inclusion in the study. HZ and ZZ performed the data extraction. HY, SY, and BY were involved in the analysis of the data. HW made good suggestions for the article. All authors read and approved the final manuscript.
