# Supplementary material for: Effect of acupuncture on the modulation of functional brain regions in migraine: A meta-analysis of fMRI studies
Source: Front Neurol. 2023 Mar 8;14:1036413. doi: 10.3389/fneur.2023.1036413 (PMC10031106; doi:10.3389/fneur.2023.1036413)
Supplement: Supplementary file 2 [file Table_2.pdf]

TableS2. Search strategies for PubMed databases

No.	Mesh
#1	Magnetic resonance imaging
#2	Functional magnetic resonance imaging
#3	neuroimaging
#4	Functional MRI
#5	Resting-state functional magnetic resonance
#6	rs-fmri
#7	#1 OR #2 OR #3 OR #4 OR #5 OR #6
#8	ALFF
#9	fALFF
#10	Amplitude of Low Frequency Fluctuations
#11	ReHo
#12	Regional homogeneity
#13	local connectivity
#14	coherence
#15	#8 OR #9 OR #10 #11 OR #12 OR #13 OR #14
#16	Acupuncture
#17	Needle
#18	Electroacupuncture
#19	Acupoint
#20	#16 OR #17 OR #18 OR #19
#21	Migraine
#22	RCT
#23	randomized controlled trials
#24	clinical trials
#25	randomized
#26	#22 OR #23 OR #24 OR #25
#27	#7 AND #15 AND #20 AND #26
