# Supplementary material for: Effect of acupuncture on the modulation of functional brain regions in migraine: A meta-analysis of fMRI studies
Source: Front Neurol. 2023 Mar 8;14:1036413. doi: 10.3389/fneur.2023.1036413 (PMC10031106; doi:10.3389/fneur.2023.1036413)
Supplement: Supplementary file 3 [file Table_3.pdf]

Table S3. Quality Assessment Checklist (1 point per criterion for fully satisfied, 0.5 for partially satisfied, 0 for otherwise)

**Category 1: Subjects**	**Score** (0/0.5/1)
1. Patients were evaluated prospectively, specific diagnostic criteria were applied, and demographic data were reported.	
2. Healthy subjects were evaluated prospectively, and psychiatric and medical illnesses were excluded.	
3. Important variables (such as age, gender, illness duration, onset time, medication status, comorbidity, and severity of illness) were checked, either by stratification or statistically.	
4. Sample size per group > 10.	
**Category 2: Methods for image acquisition and analysis**	
5. Magnet strength ≥1.5T.	
6. MRI slice thickness ≤2 mm.	
7. The whole-brain analysis was automatically calculated with no prior regional selection.	
8. Coordinates were reported in a standard space.	
9. The imaging technique processing was described clearly enough to be reproducible.	
10. Measurements were described clearly enough to be reproducible.	
**Category 3: Results and conclusions**	
11. Statistical parameters were provided.	
12. Conclusions were consistent with the results obtained and the limitations were discussed.	
**TOTAL**	/12

Table S2. Meta-regression analysis of the correlation between GM alterations and clinical variables in migraine patients using the AES-SDM method.

	MNI coordinate			SDM z-score [a]	P value [b]	Number of voxels [c]	Cluster breakdown (number of voxels)
	X	Y	Z				
**Duration of migraine**							
Bilateral cerebellum and R dorsal medulla	0	-44	-46	1.932	<0.001	1422	R cerebellum, hemispheric lobule IX (335)<br>L cerebellum, hemispheric lobule IX (170)<br>R dorsal medulla (144)
L insula	-36	-12	14	1.398	0.001	465	L insula (255)<br>L rolandic operculum (65)
**Frequency of migraine attacks**							
L rolandic operculum	-62	0	4	2.987	<0.001	273	L rolandic operculum (150)<br>L superior temporal gyrus (84)
**Age**							
R dorsal medulla	6	-34	-42	1.377	0.003	77	
L amygdala	-26	-8	-24	-1.664	<0.001	413	L amygdala (96)<br>L parahippocampus (66)<br>L hippocampus (21)
L parahippocampus	-28	-32	-18	-1.336	<0.001	169	L parahippocampus (32)
R parahippocampus	20	2	-24	-1.234	0.001	33	R parahippocampus (28)

[a] Peak height threshold: $z > 1$.

[b] Voxel probability threshold: $P < 0.005$.

[c] Cluster extent threshold: regions with < 20 voxels are not reported in the cluster breakdown.

Abbreviations: GM, gray matter; MNI, Montreal Neurological Institute; L, left; R, right; AES-SDM, anisotropic effect size-signed differential mapping.

Table S3. Subgroup analysis of GM alterations in migraine patients using the AES-SDM method.

	MNI coordinate			SDM z-score [a]	P value [b]	Number of voxels [c]	Cluster breakdown (number of voxels)
	X	Y	Z				
**MwoA > HS**							
L amygdala	-28	-4	-22	2.007	<0.001	739	L amygdala, BA28, BA34 (141)<br>L temporal pole, superior temporal gyrus, BA28, BA38 (99)<br>L parahippocampus, BA28 (43)<br>L hippocampus, BA28 (20)
R parahippocampus	20	0	-24	1.980	<0.001	577	R parahippocampus, BA28, BA34 (150)<br>R amygdala, BA34 (127)
L parahippocampus	-26	-34	-18	2.026	<0.001	428	L fusiform gyrus, BA30, BA37 (170)<br>L cerebellum, hemispheric lobule IV / V, BA30, BA37 (63)<br>L parahippocampus, BA30 (51)<br>L hippocampus, BA20 (31)
R hippocampus	32	-24	-14	1.392	0.001	90	R hippocampus, BA20 (75)
L lingual gyrus	-18	-96	-20	1.400	0.001	38	L lingual gyrus, BA 18 (23)
**MwoA < HS**							
Bilateral dorsal medulla and cerebellum	0	-40	-52	-1.504	<0.001	746	R dorsal medulla (138)<br>R cerebellum, hemispheric lobule IX (49)<br>L dorsal medulla (45)<br>L cerebellum, hemispheric lobule IX (34)
R inferior frontal gyrus	42	26	-14	-1.460	<0.001	227	R inferior frontal gyrus, orbital part, BA 38, BA 47 (175)<br>R insula, BA 47 (25)
R middle frontal gyrus	32	44	30	-1.361	0.001	48	R middle frontal gyrus, BA46 (45)
R supplementary motor area	8	22	62	-1.292	0.002	32	R supplementary motor area, BA8 (20)
**MwA > HS**							
R inferior occipital gyrus	32	-92	-6	1.013	<0.001	364	R inferior occipital gyrus, BA18, BA19 (221)<br>R middle occipital gyrus, BA18 (51)
L middle temporal gyrus	-46	-56	16	1.001	<0.001	244	L middle temporal gyrus, BA21, BA39 (185)
**MwA < HS**							
L supplementary motor area	2	20	44	-1.022	0.001	715	L superior frontal gyrus, medial, BA8, BA32 (150)<br>L supplementary motor area BA8, BA32 (148)

							R median cingulate/paracingulate gyrus, BA24, BA32 (132)<br>L median cingulate/paracingulate gyrus, BA24 (90)<br>R superior frontal gyrus, medial, BA8, BA32 (75)<br>R supplementary motor area, BA8, BA32 (73)
R cerebellum	2	-64	-56	-1.094	<0.001	79	R cerebellum, hemispheric lobule IX (24)
R superior frontal gyrus, dorsolateral	26	4	56	-1.012	0.002	35	
L temporal pole, superior temporal gyrus	-58	6	-2	-1.003	0.003	26	
**EM > HS**							
L temporal pole, superior temporal gyrus	-28	8	-24	1.777	<0.001	1125	L temporal pole, superior temporal gyrus, BA28, BA38 (280)<br>L amygdala, BA28, BA34 (91)<br>L temporal pole, middle temporal gyrus, BA20 (38)<br>L insula, BA48 (34)<br>L parahippocampus, BA28 (29)
L middle temporal gyrus	-46	-56	16	2.204	<0.001	822	L middle temporal gyrus, BA21, BA22, BA37, BA39 (582)<br>L angular gyrus, BA39 (87)<br>L superior temporal gyrus, BA22 (25)
R superior frontal gyrus	10	46	38	1.624	<0.001	375	R superior frontal gyrus, BA9, BA10, BA32 (232)
R superior temporal gyrus	44	-34	2	1.349	0.001	316	R superior temporal gyrus, BA21, BA22, BA42 (103)<br>R middle temporal gyrus, BA21, BA22 (95)
R amygdala	30	-4	-18	1.272	0.002	160	R amygdala, BA34, BA36 (76)
**EM < HS**							
Bilateral dorsal medulla and cerebellum	-8	-42	-46	-1.863	<0.001	720	R dorsal medulla (130)<br>L dorsal medulla (47)<br>L cerebellum, hemispheric lobule IX (21)
L anterior thalamic projection	-4	-16	-6	-1.281	0.002	42	
**CM > HS**							
R middle frontal gyrus	42	36	22	1.489	<0.001	457	R middle frontal gyrus, BA45, BA46 (307)<br>R inferior frontal gyrus, triangular part, BA45 (99)
R caudate nucleus	8	16	10	1.332	<0.001	349	R caudate nucleus, BA25 (148)<br>R anterior thalamic projection (87)
L rolandic operculum	-62	0	6	1.231	0.002	53	L rolandic operculum, BA48 (42)
L superior occipital gyrus	-16	-94	32	1.275	0.002	29	

**CM < HS**								
L inferior temporal gyrus	-34	-10	-44	-1.151	0.004	972	L temporal pole, middle temporal gyrus, BA20, BA36, BA38 (233)
							L inferior temporal gyrus, BA20, BA36 (197)
							L fusiform gyrus, BA20, BA36 (101)
							L temporal pole, superior temporal gyrus, BA38 (89)
R inferior frontal gyrus, orbital part	40	24	-12	-1.156	0.003	72	R inferior frontal gyrus, orbital part, BA47 (27)
R inferior temporal gyrus	40	2	-44	-1.157	0.003	41	R inferior temporal gyrus, BA20 (22)
L superior frontal gyrus, dorsolateral	-28	66	6	-1.240	0.002	30	
R superior frontal gyrus, orbital part	16	70	-8	-1.220	0.003	26	
L precentral gyrus	-34	-20	64	-1.155	0.003	26	L precentral gyrus, BA6 (25)
**VM > HS**							
L superior occipital gyrus	-20	-84	26	1.021	<0.001	1443	L superior occipital gyrus, BA18, BA19 (388)
							L middle occipital gyrus, BA18, BA19 (340)
							L cuneus cortex, BA18, BA19 (220)
**VM < HS**							
L superior temporal gyrus	-52	-6	2	-2.594	<0.001	3396	L superior temporal gyrus, BA22, BA41, BA42, BA48 (789)
							L insula, BA48 (616)
							L rolandic operculum, BA48 (489)
							L supramarginal gyrus, BA2, BA48 (272)
							L lenticular nucleus, putamen, BA48 (173)
							L heschl gyrus, BA48 (168)
							L postcentral gyrus, BA48 (83)
R superior temporal gyrus	48	-24	12	-2.514	<0.001	2683	R insula, BA48 (672)
							R rolandic operculum, BA48 (598)
							R superior temporal gyrus, BA22, BA48 (306)
							R lenticular nucleus, putamen, BA48 (214)
							R heschl gyrus, BA48 (171)
R middle frontal gyrus	42	48	16	-2.536	<0.001	566	R middle frontal gyrus, BA10, BA45, BA46 (441)
							R superior frontal gyrus, dorsolateral, BA10 (49)
**Migraine patients in interictal period > HS**							
R amygdala	24	0	-20	1.677	<0.001	1055	R parahippocampus, BA28, BA34, BA36 (229)
							R amygdala, BA34, BA36 (158)

							R temporal pole, superior temporal gyrus, BA 38 (42)
L amygdala	-26	-8	-22	1.387	<0.001	413	L amygdala, BA 28, BA 34 (107)
							L hippocampus, BA 28 (20)
L lingual gyrus	-18	-96	-20	1.184	<0.001	136	L lingual gyrus, BA18 (73)
							L inferior occipital gyrus, BA18 (35)
L hippocampus	-28	-22	-14	1.143	<0.001	133	L hippocampus, BA20 (65)
R cerebellum, hemispheric lobule III	10	-34	-12	1.025	0.001	85	R cerebellum, hemispheric lobule III, BA30 (28)
R lingual gyrus	22	-94	-20	1.099	<0.001	79	R lingual gyrus, BA18 (52)
R hippocampus	32	-22	-16	1.012	0.001	58	R hippocampus, BA20 (44)
**Migraine patients in interictal period < HS**							
L rolandic operculum	-44	-6	6	-1.962	<0.001	1120	L insula, BA48 (432)
							L rolandic operculum, BA48 (261)
							L heschl gyrus, BA48 (117)
							L superior temporal gyrus, BA48(114)
R middle frontal gyrus	36	46	26	-1.792	<0.001	291	R middle frontal gyrus, BA46 (263)
R dorsal medulla	0	-42	-50	-1.502	0.002	247	R dorsal medulla (23)
							R cerebellum, hemispheric lobule IX (22)
L cerebellum, hemispheric lobule VI	-28	-66	-22	-1.494	0.002	195	L cerebellum, hemispheric lobule VI, BA19, BA37 (182)
R inferior parietal (excluding supramarginal and angular) gyrus	46	-46	44	-1.472	0.002	80	R inferior parietal (excluding supramarginal and angular) gyrus, BA40 (64)
R rolandic operculum	54	8	2	-1.456	0.002	62	R rolandic operculum, BA48 (42)

[a] Peak height threshold: z > 1.

[b] Voxel probability threshold: P < 0.005.

[c] Cluster extent threshold: regions with < 20 voxels are not reported in the cluster breakdown.

Abbreviations: GM, gray matter; L, left; R, right; MwA, migraine with aura; MwoA, migraine without aura; HS, healthy subjects; MNI, Montreal Neurological Institute; AES-SDM, anisotropic effect size-signed differential mapping; EM, episodic migraine; CM, chronic migraine; VM, vestibular migraine.

Table S4. Subgroup analysis of GM alterations in migraine patients using the ALE method.

Cluster no.	Cluster size (mm^3)	Weighted center (x, y, z)			x	y	z	ALE value ($\times 10^{-3}$)	Label (Nearest Gray Matter within 5mm)
**MwoA > HS**									
1	720	-21.5	-31.4	-13.8	-22	-32	-14	18.222	L parahippocampus, BA35
**EM > HS**									
1	600	-19.6	-30.9	-11.1	-20	-32	-12	16.102	L parahippocampus, BA28
**VM > HS**									
1	1728				-19	-84	28	9.246	L occipital gyrus, BA18
**Migraine patients in interictal periods > HS**									
1	760	-21.6	-31.3	-13.8	-22	-32	-14	18.222	L parahippocampus, BA35

Abbreviations: GM, gray matter; ALE, activation likelihood estimation; L, left; MwoA, migraine without aura; EM, episodic migraine; VM, vestibular migraine; HS, healthy subjects; R, right.

Table S5. Heterogeneity of altered GM regions between migraine patients and HS in VBM studies using the AES-SDM method.

Regions	MNI coordinate			SDM z-score [(a)]	p-value [(b)]	Number of voxels [(c)]
	x	y	z			
L insula	-38	-10	12	2.274	<0.001	822
R middle frontal gyrus	46	40	30	2.815	<0.001	117

[a] Peak height threshold: $z > 1$;

[b] Voxel probability threshold: $p < 0.005$;

[c] Cluster threshold: Regions with less than 10 voxels are not reported.

Abbreviations: GM, gray matter; HS, healthy subjects; L, left; R, right; MNI, Montreal Neurological Institute; AES-SDM, anisotropic effect size-signed differential mapping; VBM, voxel-based morphometry.

Table S6. Sensitivity analysis of VBM meta-analysis using the AES-SDM method.

VBM studies	Increased GM regions						Decreased GM regions					
	Bilateral amygdala	Bilateral parahippocampus	L hippocampus	Bilateral temporal poles and superior temporal gyri	R superior frontal gyrus	L middle temporal gyrus	L insula	Bilateral cerebellum (hemispheric lobule IX)	R dorsal medulla	Bilateral rolandic operculum	R middle frontal gyrus	R right inferior parietal gyrus
Matharu MS 2003 [1]	Yes	Yes	Yes	Yes	Yes	Yes	Yes	Yes	Yes	Yes	Yes	Yes
Rocca MA 2006[2]	Yes	Yes	Yes	Yes	Yes	Yes	Yes	Yes	Yes	Yes	Yes	Yes
Schmitz N 2008[3]	Yes	Yes	Yes	Yes	Yes	Yes	Yes	Yes	Yes	Yes	No	Yes
Kim JH 2008[4]	Yes	Yes	Yes	Yes	Yes	Yes	Yes	Yes	Yes	Only left	No	No
Schmidt-Wilcke T 2008[5]	Yes	Yes	Yes	Yes	Yes	Yes	Yes	Yes	Yes	Yes	Yes	Yes
Tessitore A 2012[6]	Yes	Yes	Yes	Yes	Yes	Yes	Yes	Yes	Yes	Yes	Yes	Yes
Hubbard CS 2014[7]	Yes	Yes	Yes	Yes	Yes	Yes	Yes	Yes	Yes	Yes	Yes	Yes
Chanraud S 2014[8]	Yes	Yes	Yes	Yes	Yes	Yes	Yes	Yes	Yes	Yes	Yes	Yes
Obermann M 2014[9]	Yes	Yes	Yes	Yes	Yes	Yes	Yes	Yes	Yes	Yes	Yes	Yes
Tessitore A 2015[10] (MwoA)	Yes	Yes	Yes	Yes	Yes	Yes	Yes	Yes	Yes	Yes	Yes	Yes
Tessitore A 2015[10] (MwA)	Yes	Yes	Yes	Yes	Yes	Yes	Yes	Yes	Yes	Yes	Yes	Yes
Coppola G 2015[11] (Interictal)	Yes	Yes	Yes	Yes	Yes	Yes	Yes	Yes	Yes	Only left	Yes	No
Coppola G 2015[11] (Ictal)	Yes	Yes	Yes	Yes	Yes	Yes	Yes	Yes	Yes	Yes	Yes	No

Liu JX 2015[12]	Yes	Only right	No	Yes	Yes	Yes	Yes	Yes	Yes	Yes	No	Yes
Lai TH 2016[13]	Yes	Yes	Yes	Yes	Yes	Yes	Yes	Yes	Yes	Yes	Yes	Yes
Hougaard A 2016[14]	Yes	Yes	Yes	Yes	Yes	Yes	Yes	Yes	Yes	Yes	Yes	Yes
Zhang JL 2017[15]	Yes	Yes	Yes	Yes	Yes	Yes	Yes	Yes	Yes	Yes	Yes	Yes
Liu JX 2017[16]	Yes	Yes	Yes	Yes	Yes	Yes	Yes	Yes	Yes	Yes	Yes	Yes
Coppola G 2017[17]	Yes	Yes	Yes	Yes	Yes	Yes	Yes	Yes	Yes	Yes	Yes	Yes
Messina R 2017[18]	Yes	Yes	Yes	Yes	Yes	No	Yes	Yes	Yes	Yes	Yes	Yes
Neeb L 2017[19] (Episodic)	Yes	Yes	Yes	Yes	Yes	Yes	Yes	Yes	Yes	Yes	Yes	Yes
Neeb L 2017[19] (Chronic)	Yes	Yes	Yes	Yes	Yes	Yes	Yes	Yes	Yes	Yes	Yes	Yes
Arkink EB 2017[20] (MwoA)	Yes	Yes	Yes	Yes	Yes	Yes	Yes	Yes	Yes	Yes	Yes	Yes
Arkink EB 2017[20] (MwA)	Yes	Yes	Yes	Yes	Yes	Yes	Yes	Yes	Yes	Yes	Yes	Yes
Palm-Meinders IH 2017[21]	Yes	Yes	Yes	Yes	No	No	Yes	Yes	Yes	Yes	Yes	Yes
Chen WT 2018[22]	Yes	Yes	Yes	Yes	Yes	Yes	Yes	Yes	Yes	Yes	Yes	Yes
Celle S 2018[23]	Yes	Yes	Yes	Yes	Yes	Yes	Yes	Yes	Yes	Yes	Yes	Yes
Messina R 2018[24]	Yes	Yes	Yes	Yes	Yes	No	Yes	Yes	Yes	Yes	Yes	Yes
Husøy AK 2019[25]	Yes	Yes	Yes	Yes	Yes	Yes	Yes	Yes	Yes	Yes	Yes	Yes
Wei HL 2019[26]	Yes	Yes	Yes	Yes	Yes	Yes	Yes	Yes	Yes	Yes	Yes	Yes
Yang FC 2019[27]	Yes	Yes	Yes	Yes	Yes	Yes	Yes	Yes	Yes	Yes	Yes	Yes

Bonanno L 2020[28] (MwoA)	Yes	Yes	Yes	Yes	Yes	Yes	Yes	Yes	Yes	Yes	No	Yes
Bonanno L 2020[28] (MwA)	Yes	Yes	Yes	Yes	Yes	Yes	Yes	Yes	Yes	Yes	Yes	Yes
Li ZJ 2020[29]	Yes	Yes	Yes	Yes	Yes	Yes	Yes	No	No	Yes	Yes	Yes
Liu HY 2020[30]	Yes	Yes	Yes	Yes	Yes	Yes	Yes	Yes	Yes	Yes	Yes	No
Zhe X 2021[31]	Yes	Yes	Yes	Yes	Yes	Yes	No	Yes	Yes	No	Yes	Yes
Yu Y 2021[32] (Episodic)	Yes	Yes	Yes	Yes	Yes	Yes	Yes	Yes	Yes	Yes	No	No
Yu Y 2021[32] (Chronic)	Yes	Yes	Yes	Yes	Yes	Yes	Yes	Yes	Yes	Yes	Yes	Yes
Chou KH 2021[33]	Yes	Yes	Yes	Yes	Yes	Yes	Yes	Yes	Yes	Yes	Yes	Yes
Masson R 2021[34]	Yes	Yes	Yes	Yes	Yes	Yes	Yes	Yes	Yes	Yes	Yes	Yes
Zhao L 2011[35]	Yes	Yes	Yes	Yes	Yes	Yes	Yes	Yes	Yes	Only left	Yes	Yes
Chen XY 2014[36] (Episodic)	Yes	Yes	Yes	Yes	No	Yes	Yes	Yes	Yes	Yes	Yes	No
Chen XY 2014[36] (Chronic)	Yes	Yes	Yes	Yes	Yes	Yes	Yes	Yes	Yes	Yes	Yes	Yes
Yao Q 2017[37]	Yes	Yes	Yes	Yes	Yes	Yes	Yes	Yes	Yes	Yes	Yes	Yes
Zhe X 2018[38]	Yes	Yes	Yes	Yes	Yes	Yes	No	Yes	Yes	Only right	No	No
Li MQ 2020[39]	Yes	Yes	Yes	Yes	Yes	Yes	Yes	Yes	Yes	Yes	Yes	Yes
Wang JH 2021[40]	Yes	Yes	Yes	Yes	Yes	Yes	Yes	Yes	Yes	Only left	Yes	Yes
	47/47	46/47	46/47	47/47	45/47	44/47	45/47	46/47	46/47	41/47	41/47	40/47

Abbreviations: VBM, voxel-based morphometry; AES-SDM, anisotropic effect size-signed differential mapping; L, left; R, right; GM, gray

matter.

Figure S1. Results of funnel plot analysis for the meta-analysis of all included VBM studies using the AES-SDM method.

The Egger's test and funnel plots revealed no significant publication bias in the (A) right amygdala (Z=-0.31, t=-0.78, df=45, P=0.441), (B) right superior frontal gyrus (Z=0.10, t=0.22, df=45, P=0.826), (C) left hippocampus (Z=-0.53, t=-1.98, df=45, P=0.054), (D) left middle temporal gyrus (Z=-0.19, t=-0.57, df=45, P=0.570), (E) bilateral cerebellum and right dorsal medulla (Z=-0.06, t=-0.13, df=45, P=0.894), (F) left insula (Z=-1.06, t=-1.79, df=45, P=0.080), (G) right rolandic operculum (Z=-0.58, t=-1.46, df=45, P=0.151), (H) right middle frontal gyrus (Z=-0.64, t=-1.63, df=45, P=0.110) and (I) right inferior parietal gyrus (Z=-0.53, t=-1.38, df=45, P=0.175). Significant publication bias was reported in the (J) left amygdala (Z=-0.76, t=-2.44, df=45, P=0.019) by the Egger's test and funnel plots.

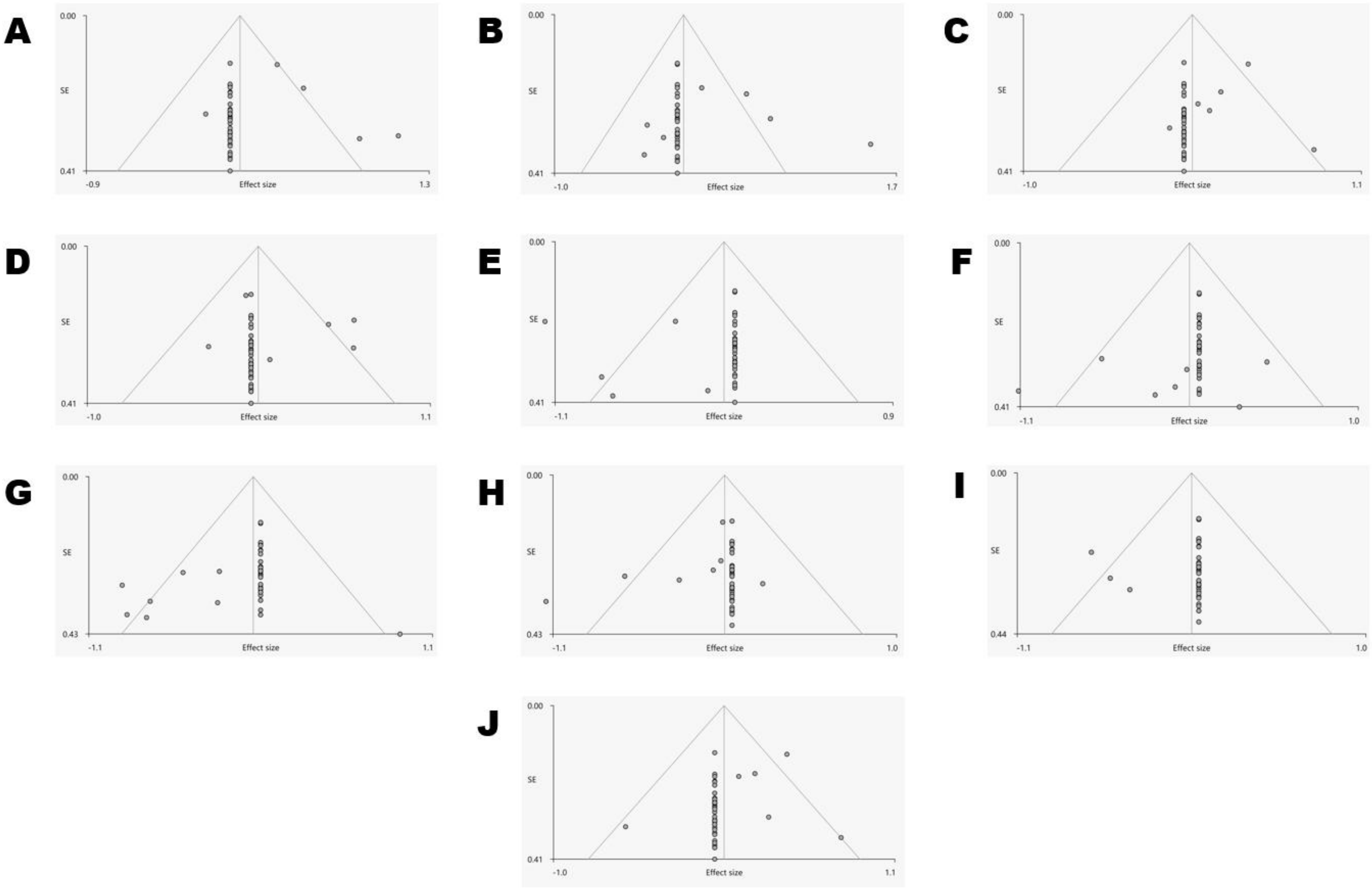

Abbreviations: VBM, voxel-based morphometry; SE, standard error; AES-SDM, anisotropic effect size-signed differential mapping.

Reference

1. Matharu MS, Good CD, May A et al (2003) No change in the structure of the brain in migraine: a voxel-based morphometric study. Eur J Neurol 10(1):53-57. https://doi.org/10.1046/j.1468-1331.2003.00510.x
2. Rocca MA, Ceccarelli A, Falini A et al (2006) Brain gray matter changes in migraine patients with T2-visible lesions: a 3-T MRI study. Stroke 37(7):1765-1770. https://doi.org/10.1161/01.STR.0000226589.00599.4d
3. Schmitz N, Admiraal-Behloul F, Arkink EB et al (2008) Attack frequency and disease duration as indicators for brain damage in migraine. Headache 48(7):1044-1055. https://doi.org/10.1111/j.1526-4610.2008.01133.x
4. Kim JH, Suh SI, Seol HY et al (2008) Regional grey matter changes in patients with migraine: a voxel-based morphometry study. Cephalalgia 28(6):598-604. https://doi.org/10.1111/j.1468-2982.2008.01550.x
5. Schmidt-Wilcke T, Gänssbauer S, Neuner T et al (2008) Subtle grey matter changes between migraine patients and healthy controls. Cephalalgia 28(1):1-4. https://doi.org/10.1111/j.1468-2982.2007.01428.x
6. Tessitore A, Russo A, Giordano A et al (2013) Disrupted default mode network connectivity in migraine without aura. J Headache Pain 14(1):89. https://doi.org/10.1186/1129-2377-14-89
7. Hubbard CS, Khan SA, Keaser ML et al (2014) Altered Brain Structure and Function Correlate with Disease Severity and Pain Catastrophizing in Migraine Patients. eNeuro 1(1):e20.14. https://doi.org/10.1523/ENEURO.0006-14.2014
8. Chanraud S, Di Scala G, Dilharreguy B et al (2014) Brain functional connectivity and morphology changes in medication-overuse headache: Clue for dependence-related processes? Cephalalgia 34(8):605-615. https://doi.org/10.1177/0333102413519514
9. Obermann M, Wurthmann S, Steinberg BS et al (2014) Central vestibular system modulation in vestibular migraine. Cephalalgia 34(13):1053-1061. https://doi.org/10.1177/0333102414527650
10. Tessitore A, Russo A, Conte F et al (2015) Abnormal Connectivity Within Executive Resting-State Network in Migraine With Aura. Headache 55(6):794-805. https://doi.org/10.1111/head.12587
11. Coppola G, Di Renzo A, Tinelli E et al (2015) Evidence for brain morphometric changes during the migraine cycle: a magnetic resonance-based morphometry study. Cephalalgia 35(9):783-791. https://doi.org/10.1177/0333102414559732
12. Liu J, Lan L, Mu J et al (2015) Genetic contribution of catechol-O-methyltransferase in hippocampal structural and functional changes of female migraine sufferers. Hum Brain Mapp 36(5):1782-1795. https://doi.org/10.1002/hbm.22737

13. Lai TH, Chou KH, Fuh JL et al (2016) Gray matter changes related to medication overuse in patients with chronic migraine. Cephalalgia 36(14):1324-1333. https://doi.org/10.1177/0333102416630593
14. Hougaard A, Amin FM, Arngrim N et al (2016) Sensory migraine aura is not associated with structural grey matter abnormalities. Neuroimage Clin 11:322-327. https://doi.org/10.1016/j.nicl.2016.02.007
15. Zhang J, Wu YL, Su J et al (2017) Assessment of gray and white matter structural alterations in migraineurs without aura. J Headache Pain 18(1):74. https://doi.org/10.1186/s10194-017-0783-5
16. Liu J, Mu J, Liu Q et al (2017) Brain structural properties predict psychologically mediated hypoalgesia in an 8-week sham acupuncture treatment for migraine. Hum Brain Mapp 38(9):4386-4397. https://doi.org/10.1002/hbm.23667
17. Coppola G, Petolicchio B, Di Renzo A et al (2017) Cerebral gray matter volume in patients with chronic migraine: correlations with clinical features. J Headache Pain 18(1):115. https://doi.org/10.1186/s10194-017-0825-z
18. Messina R, Rocca MA, Colombo B et al (2017) Structural brain abnormalities in patients with vestibular migraine. J Neurol 264(2):295-303. https://doi.org/10.1007/s00415-016-8349-z
19. Neeb L, Bastian K, Villringer K et al (2017) Structural Gray Matter Alterations in Chronic Migraine: Implications for a Progressive Disease? Headache 57(3):400-416. https://doi.org/10.1111/head.13012
20. Arkink EB, Schmitz N, Schoonman GG et al (2017) The anterior hypothalamus in cluster headache. Cephalalgia 37(11):1039-1050. https://doi.org/10.1177/0333102416660550
21. Palm-Meinders IH, Arkink EB, Koppen H et al (2017) Volumetric brain changes in migraineurs from the general population. Neurology 89(20):2066-2074. https://doi.org/10.1212/WNL.0000000000004640
22. Chen WT, Chou KH, Lee PL et al (2018) Comparison of gray matter volume between migraine and "strict-criteria" tension-type headache. J Headache Pain 19(1):4. https://doi.org/10.1186/s10194-018-0834-6
23. Celle S, Créac'h C, Boutet C et al (2018) Elderly Patients with Ongoing Migraine Show Reduced Gray Matter Volume in Second Somatosensory Cortex. J Oral Facial Pain Headache 32(1):67–74. https://doi.org/10.11607/ofph.1866
24. Messina R, Rocca MA, Colombo B et al (2018) Gray matter volume modifications in migraine: A cross-sectional and longitudinal study. Neurology 91(3):e280-e292. https://doi.org/10.1212/WNL.0000000000005819
25. Husøy AK, Håberg AK, Rimol LM et al (2019) Cerebral cortical dimensions in headache sufferers aged 50 to 66 years: a population-based imaging study in the Nord-Trøndelag Health Study (HUNT-MRI). Pain 160(7):1634-1643.

https://doi.org/10.1097/j.pain.0000000000001550
26. Wei HL, Zhou X, Chen YC et al (2019) Impaired intrinsic functional connectivity between the thalamus and visual cortex in migraine without aura. J Headache Pain 20(1):116. https://doi.org/10.1186/s10194-019-1065-1
27. Yang FC, Chou KH, Lee PL et al (2019) Patterns of gray matter alterations in migraine and restless legs syndrome. Ann Clin Transl Neurol 6(1):57-67. https://doi.org/10.1002/acn3.680
28. Bonanno L, Lo Buono V, De Salvo S et al (2020) Brain morphologic abnormalities in migraine patients: an observational study. J Headache Pain 21(1):39. https://doi.org/10.1186/s10194-020-01109-2
29. Li Z, Zhou J, Lan L et al (2020) Concurrent brain structural and functional alterations in patients with migraine without aura: an fMRI study. J Headache Pain 21(1):141. https://doi.org/10.1186/s10194-020-01203-5
30. Liu HY, Lee PL, Chou KH et al (2020) The cerebellum is associated with 2-year prognosis in patients with high-frequency migraine. J Headache Pain 21(1):29. https://doi.org/10.1186/s10194-020-01096-4
31. Zhe X, Zhang X, Chen L et al (2021) Altered Gray Matter Volume and Functional Connectivity in Patients With Vestibular Migraine. Front Neurosci 15:683802. https://doi.org/10.3389/fnins.2021.683802
32. Yu Y, Zhao H, Dai L et al (2021) Headache frequency associates with brain microstructure changes in patients with migraine without aura. Brain Imaging Behav 15(1):60-67. https://doi.org/10.1007/s11682-019-00232-2
33. Chou KH, Lee PL, Liang CS et al (2021) Identifying neuroanatomical signatures in insomnia and migraine comorbidity. Sleep 44(3). https://doi.org/10.1093/sleep/zsaa202
34. Masson R, Demarquay G, Meunier D et al (2021) Is Migraine Associated to Brain Anatomical Alterations? New Data and Coordinate-Based Meta-analysis. Brain Topogr 34(3):384-401. https://doi.org/10.1007/s10548-021-00824-6
35. Zhao L (2011) Functional Connectivity Network involved in Acupuncture Along Meridians based on fMRI Study. Chengdu University of Traditional Chinese Medicine.
36. Chen X (2014) Chronification of Migraine: a Clinical and Brain Gray Matter Structure Study. Chinese PLA Medical School.
37. Yao Q (2017) Grey matter volume abnormality affected by mood disorder in migraine without aura - initial exploration. Shanghai Jiao Tong University.
38. Zhe X, Zhang X, Chen L et al (2018) Cerebral grey matter volume abnormalities in patients with vestibular migraine. Diagnostic imaging and interventional radiology 27(6):428-432.

39. Li M, Li X, Zhu W et al (2020) The study of correlations between structural changes of gray matter and cognitive decline in patients with migraine without aura. Radiologic practice 35(3):329-333.
40. Wang J, Liu B, Yu D et al (2021) Voxel-based gray matter volume study in patients with vestibular migraine. Chinese journal of magnetic resonance imaging 12(3):67-70+88.
