# Supplementary material for: Effect of acupuncture on the modulation of functional brain regions in migraine: A meta-analysis of fMRI studies
Source: Front Neurol. 2023 Mar 8;14:1036413. doi: 10.3389/fneur.2023.1036413 (PMC10031106; doi:10.3389/fneur.2023.1036413)
Supplement: Supplementary file 4 [file Table_4.pdf]

S4. Anatomical localization of the acupoints mentioned in the included studies

1.GB41 (Zulinqi): Located lateral to the dorsum of the foot, behind the 4th toe segment (4th toe joint), at the lateral recess of the extensor digitorum tendon.
2.GB44:(Zuqiaoyin): Outside the 4th toe distal, 0.1 inch from toenail angle.
3.ST45(Lidui): Outside the second unsegmented toe, 0.1 inch from toenail angle.
4.BL67(Zhiyin): Outside of distal segment of little toe, 0.1 inch from toenail angle.
5.LR1(Dadun): Outside big toe distal, 0.1 inch from toenail angle.
6.SJ5(Waiguan): Located two inches on the wrist of the wrist, in the gap between the ulna and the radius.
7.GB34(Yanglingquan): Lateral side of lower leg, depression anterior inferior of fibular head.
8.GB40(Qiuxu): Anterior inferior lateral malleolus, recessed lateral toe growth tendon.
9.ST36(Zusanli): On the medial side of the lower leg, when the tip of the inner ankle of the foot is 3 cun above, behind the medial edge of the tibia.
10.ST42(Chongyang): Located at the highest point of the dorsum of the foot, between the extensor pollicis longus tendon and the extensor digitorum longus tendon, at the pulsation of the dorsalis pedis artery
11.LI6(Pianli): Located outside the human arm, elbow flexion, 1 inch above Quchi.
Note: "cun" is the traditional Chinese unit of length, 1 cun = 3.33 cm
